# Protocatechuic acid and quercetin attenuate ETEC-caused IPEC-1 cell inflammation and injury associated with inhibition of necroptosis and pyroptosis signaling pathways

**DOI:** 10.1186/s40104-022-00816-x

**Published:** 2023-02-01

**Authors:** Kan Xiao, Mohan Zhou, Qingqing Lv, Pengwei He, Xu Qin, Dan Wang, Jiangchao Zhao, Yulan Liu

**Affiliations:** 1 Hubei Key Laboratory of Animal Nutrition and Feed Science, Wuhan Polytechnic University, Wuhan, 430023 People’s Republic of China; 2 Department of Animal Science, Division of Agriculture, University of Arkansas, Fayetteville, AR 72701 USA

**Keywords:** Cell damage, ETEC K88, Intestinal inflammation, Necroptosis, Protocatechuic acid, Pyroptosis, Quercetin

## Abstract

**Background:**

Necroptosis and pyroptosis are newly identified forms of programmed cell death, which play a vital role in development of many gastrointestinal disorders. Although plant polyphenols have been reported to protect intestinal health, it is still unclear whether there is a beneficial role of plant polyphenols in modulating necroptosis and pyroptosis in intestinal porcine epithelial cell line (IPEC-1) infected with enterotoxigenic *Escherichia coli* (ETEC) K88. This research was conducted to explore whether plant polyphenols including protocatechuic acid (PCA) and quercetin (Que), attenuated inflammation and injury of IPEC-1 caused by ETEC K88 through regulating necroptosis and pyroptosis signaling pathways.

**Methods:**

IPEC-1 cells were treated with PCA (40 μmol/L) or Que (10 μmol/L) in the presence or absence of ETEC K88.

**Results:**

PCA and Que decreased ETEC K88 adhesion and endotoxin level (*P* < 0.05) in cell supernatant. PCA and Que increased cell number (*P* < 0.001) and decreased lactate dehydrogenases (LDH) activity (*P* < 0.05) in cell supernatant after ETEC infection. PCA and Que improved transepithelial electrical resistance (TEER) (*P* < 0.001) and reduced fluorescein isothiocyanate-labeled dextran (FD4) flux (*P* < 0.001), and enhanced membrane protein abundance of occludin, claudin-1 and ZO-1 (*P* < 0.05), and rescued distribution of these tight junction proteins (*P* < 0.05) after ETEC infection. PCA and Que also declined cell necrosis ratio (*P* < 0.05). PCA and Que reduced mRNA abundance and concentration of tumor necrosis factor-α (TNF-α), interleukin (IL)-6 and IL-8 (*P* < 0.001), and down-regulated gene expression of toll-like receptors 4 (*TLR4*) and its downstream signals (*P* < 0.001) after ETEC infection. PCA and Que down-regulated protein abundance of total receptor interacting protein kinase 1 (t-RIP1), phosphorylated-RIP1 (p-RIP1), p-RIP1/t-RIP1, t-RIP3, p-RIP3, mixed lineage kinase domain-like protein (MLKL), p-MLKL, dynamin- related protein 1 (DRP1), phosphoglycerate mutase 5 (PGAM5) and high mobility group box 1 (HMGB1) (*P* < 0.05) after ETEC infection. Moreover, PCA and Que reduced protein abundance of nod-like receptor protein 3 (NLRP3), nod-like receptors family CARD domain-containing protein 4 (NLRC4), apoptosis-associated speck-like protein containing a CARD (ASC), gasdermin D (GSDMD) and caspase-1 (*P* < 0.05) after ETEC infection.

**Conclusions:**

In general, our data suggest that PCA and Que are capable of attenuating ETEC-caused intestinal inflammation and damage via inhibiting necroptosis and pyroptosis signaling pathways.

**Supplementary Information:**

The online version contains supplementary material available at 10.1186/s40104-022-00816-x.

## Introduction

Enterotoxigenic *Escherichia coli* (ETEC) is one of the leading causes of diarrhea among newborn animals, which contributes to high incidence of morbidity and mortality worldwide [1]. These microbes can adhere to intestinal epithelium cells and secrete enterotoxins impairing enterocyte functions such as increasing fluid secretion and reducing water absorption, which finally lead to the breakdown of intestinal integrity and epithelial function [2]. It is widely known that ETEC can trigger local or systemic immune response, and cause the excessive release of inflammation-related cytokines, which damages the cells or tissues [3].

Plant polyphenols are widely found in various fruits and vegetables, which has been regarded as dietary antioxidants in food products [4]. Emerging evidence has showed that plant polyphenols have various biological functions such as anti-inflammatory, anti-virus, anti-oxidative, and anti-bacterial activities [5]. Recently, protocatechuic acid (PCA, C_7_H_6_O_4_) and quercetin (Que, C_15_H_10_O_7_), the two monomeric phenols, have received great interest for researchers [6, 7]. PCA and Que are natural polyphenolic compounds as secondary metabolites from a variety of vegetables, fruits and herbs. Their structural variations contribute to their specific beneficial effects on human health [6, 7]. Que, a kind of polyhydroxy flavone, named 3,3′, 4′, 5,7-pentahydroxy flavone, is one of the most extensively distributed flavonoids in vegetables and fruits [8, 9]. Dietary supplementation of Que has been reported to attenuate intestinal damage and inflammation in piglets during long-distance transportation [10]. PCA, a kind of phenolic acid known as 3,4-dihydroxy benzoic acid, a major metabolite of anthocyanin, has been reported to increase intestinal barrier function and improve intestinal health in broilers [11]. Our previous studies have found that polyphenols from holy (HPE) (Ilex latifolia Thunb), including PCA and Que, could regulate intestinal damage induced by oxidative stress in piglets [12], however the molecular mechanisms are still little known.

Necroptosis and pyroptosis are newly identified forms of cell death, which contribute to the pathogenesis of many diseases [13, 14]. Necroptosis and pyroptosis are both tightly regulated inflammatory form of cell death, accompanied by the spread of inflammation [15]. Necroptosis morphologically exhibits the features of necrosis; however, meanwhile it exhibits a unique signaling pathway that requires involvement of receptor interaction protein kinase (RIP) 1 and RIP3, and mixed lineage kinase domain-like protein (MLKL) [16]. Pyroptosis is a form of lytic programmed cell death initiated by inflammasomes and then drives activation of caspase-1 or caspase-11/4/5, and cleaved gasdermin D (GSDMD) [17]. Currently, necroptosis and pyroptosis have been verified to play an important role in gut homeostasis and inflammation caused by multiple factors [18–21]. Until now, there is little research about the effects of plant polyphenols on necroptosis and pyroptosis signaling pathways.

Therefore, we hypothesized that PCA or Que could attenuate intestinal inflammation and damage via suppressing necroptosis and pyroptosis signaling pathways. The aim of this study was to investigate the beneficial role of PCA or Que on cell injury caused by ETEC infection and further explore molecular mechanisms.

## Materials and methods

### Cell culture

The IPEC-1 cell line was derived from mid-jejunum of a neonatal piglet, which was from Texas A&M University. IPEC-1 cells have high susceptibility to ETEC K88 infection [20]. Cells were cultured according to our previous study described [22]. PCA or Que were gained from Sigma Chemical (St. Louis, Missouri, USA).

### Bacterial strains

ETEC K88, the most prevalent ETEC strain in animal production, was applied to induce intestinal damage. ETEC K88 strain was gained from feces of piglets infected with post-weaning diarrhea, which was purchased from the China Veterinary Culture Collection Center (CVCC, Beijing, China) and cultured in Luria-Bertani (LB) medium from Oxoid (Basingstoke, Hampshire, UK) according to our previous protocols [23].

### Bacterial adhesion

Cells were inoculated on 6-well plates (Corning, NY, USA) at a density of 1 × 10^5^ cells/mL and pretreated with PCA (40 μmol/L) or Que (10 μmol/L) for 24 h and then infected with or without 1 × 10^8^ ETEC K88/mL for 3 h. The concentration of PCA and Que were determined based on our preliminary research. The bacterial adhesion was monitored at 1, 2 and 3 h post ETEC K88 infection by gradient dilution plate count method as described in previous study [23].

### Antibacterial activity

The in vitro antibacterial activity of PCA and Que against ETEC K88 was evaluated by agarose diffusion method. The bacterial growth was measured by the gradient dilution plate count method as described by [24] and finally calculated by counting the bacterial colonies. After determination of colony forming units, ETEC K88 were inoculated in LB medium contained 1% agarose for 24 h, and then oxford cups were put in in the test dish. After pouring the agar medium into the dish for solidification, the oxford cup was taken out. After that, 0, 40 μmol/L PCA or 10 μmol/L Que were added into the holes for 24 h in 37 °C as described by our previous protocols [23]. After 24 h incubation, zone of inhibition was observed to evaluate the antibacterial activity of PCA and Que compared with the positive control of antibiotics.

### Lactate dehydrogenases (LDH) activity

Cells were inoculated on 12-well plates at a density of 1 × 10^5^ cells/mL and pretreated with 0, 40 μmol/L PCA or 10 μmol/L Que for 24 h and then infected with or without 1 × 10^8^ ETEC K88/mL for 2 h. After that, cell supernatants were collected for LDH activity measurement by using a LDH assay kit (Nanjing Jiancheng Institute of Bioengineering, Nanjing, China) according to the manufacturer’s protocol. The absorbance was read at a wavelength of 450 nm using an automated microplate reader (Bio-Rad, California, USA).

### Inflammatory markers

Cells were inoculated on 6-well plates at a density of 1 × 10^5^ cells/mL and pretreated with 0, 40 μmol/L PCA or 10 μmol/L Que for 24 h and then infected with or without 1 × 10^8^ ETEC K88/mL for 2 h. The contents of TNF-α (CSE0005–096, 4A Biotech, Beijing, China), IL-6 (CSE0006–096, 4A Biotech, Beijing, China), IL-8 (CSE0008–096, 4A Biotech, Beijing, China), high mobility group box 1 (HMGB1, CSB-EL010553PI, 4A Biotech, Beijing, China) and endotoxin in cell supernatants were detected by commercially ELISA kits according to the instructions. After preparing the work solutions, standard and target sample were added into the wells and then biotinylated antibody working solution was added to react for 120 min. After that, enzyme conjugate working solution was added to maintain 30 min and then chromogenic agent was added to react for 20 min until stop solution was added. Finally, the OD450 was detected in fluorescence microplate reader and convert to the concentration of inflammatory markers, respectively (FLx800, Bio-Tek Instruments Inc., Winooski, VT, USA).

### Cell barrier function

Cells were inoculated on 12-well transwell chambers (Corning, NY, USA) at a density of 1 × 10^5^ cells/mL and then pretreated with 0, 40 μmol/L PCA or 10 μmol/L Que for 24 h, and finally infected with or without 1 × 10^8^ ETEC K88/mL for 3 h. Transepithelial electrical resistance (TEER) was measured by an EVOM voltohmmeter (Millipore, Boston, USA) as previously described [22].

The flux of FD4 was measured by flux from apical chamber to basal chamber every 12 h after ETEC K88 infection. The FD4 flux was calculated according to our previous protocols [22].

### Tight junction proteins

Cells were inoculated on confocal coverslips (Corning, NY, USA) at a density of 1 × 10^5^ cells/mL and pretreated with 0, 40 μmol/L PCA or 10 μmol/L Que for 24 h, and afterwards infected with or without 1 × 10^8^ ETEC K88/mL for another 2 h. After fixation, penetration, and blocking, primary antibodies including anti-claudin-1 (Invitrogen, Carlsbad, California, USA), occludin (Abcam, Boston, Massachusetts, USA) and ZO-1 (Biorbyt, Cambridge, UK) and secondary antibody (Invitrogen, Carlsbad, California, USA), and 4,6-diamidino-2-phenylindole (Sigma-Aldrich, St. Louis, Missouri, USA) were incubated according to our previous study [22, 23]. Confocal laser scanning microscope was used to observe distribution of tight junction proteins (Olympus FV101, Tokyo, Japan).

### Cell necrosis

Cells were inoculated on 24-well plates at a density of 1 × 10^5^ cells/mL, and afterwards pretreated with 0, 40 μmol/L PCA or 10 μmol/L Que with and without 1 × 10^8^ ETEC K88/mL infection. Cells necrosis were measured by IncuCyte ZOOM™ Live Cell Imaging System (Essen BioScience, Michigan, USA). Yoyo-3 dye was added into cells to stain necrotic cells. The data was acquired and analyzed by IncuCyte S3 software (Essen Bioscience, Michigan, USA).

### mRNA expression of inflammatory cytokines and TLR4 signals

Cells were cultured in 12-well plates at a density of 1 × 10^5^ cells/mL and pretreated with 0, 40 μmol/L PCA or 10 μmol/L Que for 24 h, and then infected with or without 1 × 10^8^ ETEC K88/mL for another 2 h. Inflammatory cytokines and TLR4 signals mRNA were measured by real-time PCR method. Firstly, total RNA was extracted using the RNAiso Plus Kit (TaKaRa Biotechnology, Beijing, China) according to the manufacturer’s guidelines. After purification and quantitation, reverse transcription was performed using the PrimeScript® RT Reagent Kit (TaKaRa Biotechnology, Beijing, China) following the manufacturer’s instructions. Quantitative analysis of the PCR was carried out on the Applied Biosystems 7500 Real-Time PCR System (Applied Biosystems, Waltham, Massachusetts, USA) using a SYBR Premix Ex Taqe (Tli Rnase H Plus) real time-PCR kit (TaKaRa Biotechnology, Beijing, China). The gene expression was calculated by the 2^-ΔΔCt^ method according to our previous protocol [23]. Expression levels of targeted biological triplicates were normalized to the reference genes *β-actin*. Primers used for real time-PCR analyses are listed in Additional file 1.

### Protein expression of necroptosis and pyroptosis signals

Cells were inoculated on 6-well plates at a density of 1 × 10^5^ cells/mL and pretreated with 0, 40 μmol/L PCA or 10 μmol/L Que for 24 h, and after that infected with or without 1 × 10^8^ ETEC K88/mL for another 2 h. Necroptosis and pyroptosis protein expression were evaluated by Western Botting.

After lysis and centrifugation, the membrane proteins and total proteins were extracted based on kit’s the procedures [22]. In this progress, primary antibodies were anti-occludin (1:1000, Abcam, Boston, Massachusetts, USA), claudin-1 (1:1000, Invitrogen, Carlsbad, California, USA), ZO-1 (1:1000, Biorbyt, Cambridge, UK), RIP1 (1:1000, LifeSpan BioSciences, Seattle, Washington, USA), p-RIP1 (1:2000, Cell Signaling Technology, Boston, Massachusetts, USA), RIP3 (1:1000, Santa Cruz Biotechnology, Santa Cruz, CA, USA), p-RIP3 (1:2000, Cell Signaling Technology), MLKL (1:1000, Cell Signaling Technology), p-MLKL (1:1000, Cell Signaling Technology), DRP1 (1:1000, Abcam, Boston, Massachusetts, USA), phosphoglycerate mutase 5 (PGAM5, 1:1000, Abcam, Boston, Massachusetts, USA), apoptosis-associated speck-like protein containing a CARD (ASC, 1:1000, Absin, Shanghai, China), nod-like receptors family CARD domain-containing protein 4 (NLRC4, 1:1000, Absin), nod-like receptor protein 3 (NLRP3, 1:1000, Novus, Littleton, Colorado, USA), GSDMD (1:1000, Affinity Biosciences, New Jersey, USA), β-actin (1:10000, Sigma Aldrich, St. Louis, Missouri, USA) and NaK-ATPase (1:1000, Cell Signaling Technology). Secondary antibody was HRP-conjugated secondary antibody (1:5000, AntGene Biotech, Wuhan, China). After that enhanced chemiluminescence kit (Amersham, Piscataway, New Jersey, USA) was used to detect and visualize blots. The relative abundance of target protein was expressed as the target protein:β-actin ratio, except for the tight junction proteins were expressed as the target protein:β-actin ratio:NaK-ATPase ratio. The phosphorylated proteins were normalized with relative total protein abundance.

### Statistical analysis

The data of ETEC K88 adherence and antibacterial activity were evaluated by student’s *t*-test. Other results were performed using the general linear model procedures of SPSS version 23 (SPSS Inc., Chicago, IL, USA) for 2 × 3 factors design. The linear model for the 2 × 3 factors design contained HPE (Control, PCA or Que), ETEC K88 (PBS or ETEC K88), and their interactions (HPE × ETEC K88). When a significant interaction or a trend for interaction was observed, post hoc testing was conduct by Duncan’s multiple comparison tests. All data were showed as means with standard errors. *P* ≤ 0.05 was set as significant, as well as 0.05 < *P* ≤ 0.10 was set as a trend.

## Results

### PCA and Que decrease ETEC K88 adhesion and endotoxin level in IPEC-1 cells

**Fig. 1:**
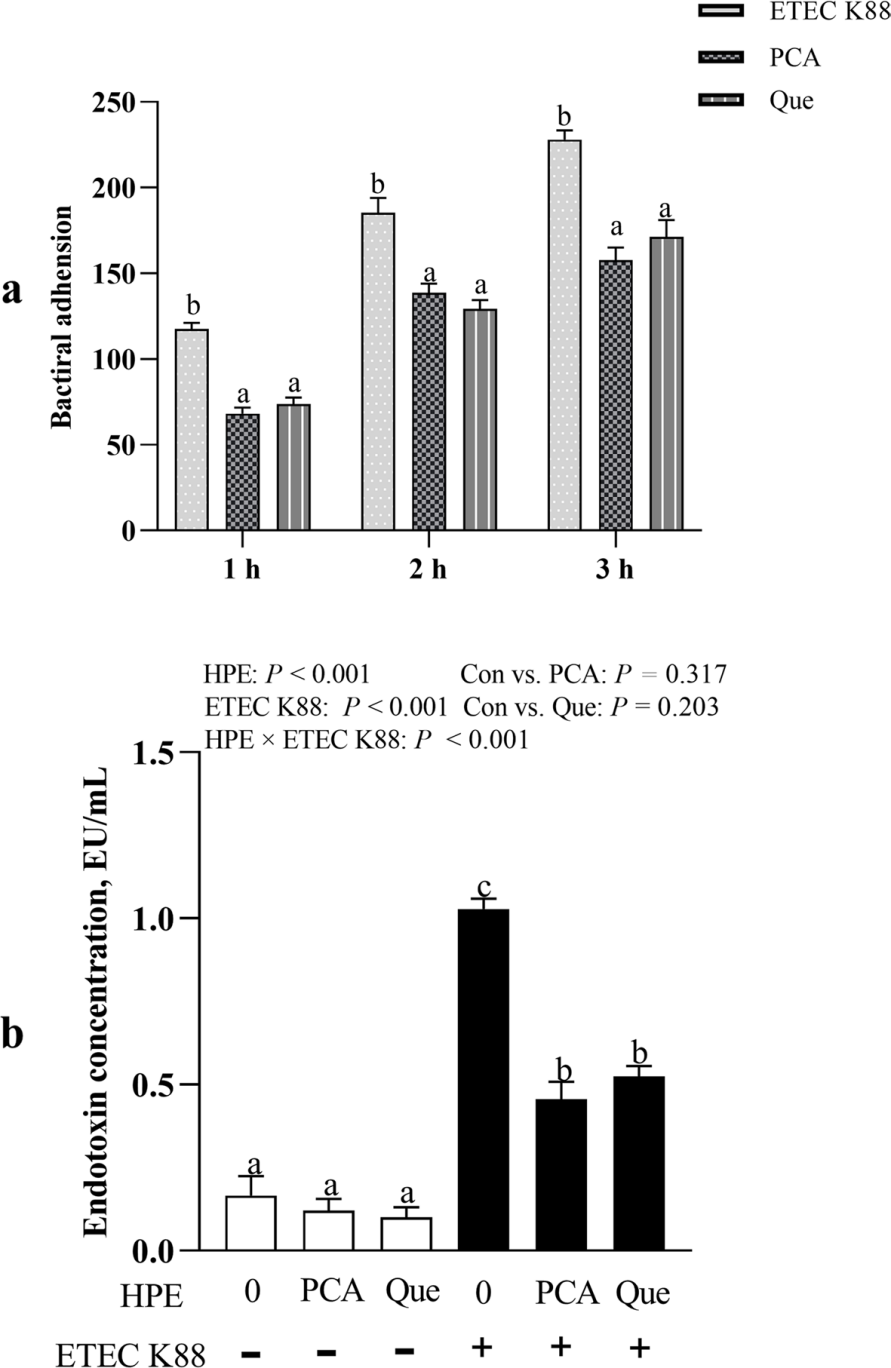
Effects of PCA and Que on ETEC K88 adhesion and endotoxin level after infection with ETEC K88 in IPEC-1 cells. Cells were pretreated with 40 μmol/L PCA or 10 μmol/L Que for 24 h and then infected with or without 1 × 10^8^ ETEC K88/mL for 2 h. **a** ETEC K88 adhesion. **b** Endotoxin level. Values are means ± SE, *n* = 6. ^a-c^Different letters represent a significant difference, *P <* 0.05. IPEC-1, intestinal porcine epithelial cell 1

PCA or Que treatment did not inhibit ETEC K88 growth (Additional file 2). Pretreating with PCA or Que decreased bacterial adhesion compared with control group at 1 h (*P* < 0.001), 2 h (*P* < 0.01) and 3 h (*P* < 0.01) after infection with ETEC K88 (Fig. 1a). ETEC K88 infection increased the endotoxin level (*P* < 0.001) in cell supernatant at 2 h (Fig. 1b). A HPE× ETEC K88 interaction (*P* < 0.001) was found for endotoxin level in which pretreating with PCA or Que decreased endotoxin secretion (*P* < 0.05) compared with control cells after infection with ETEC K88, however, there was no difference among non-ETEC K88-infected cells.

### PCA and Que increase cell number and decrease LDH activity after infection with ETEC K88 in IPEC-1 cells

**Fig. 2:**
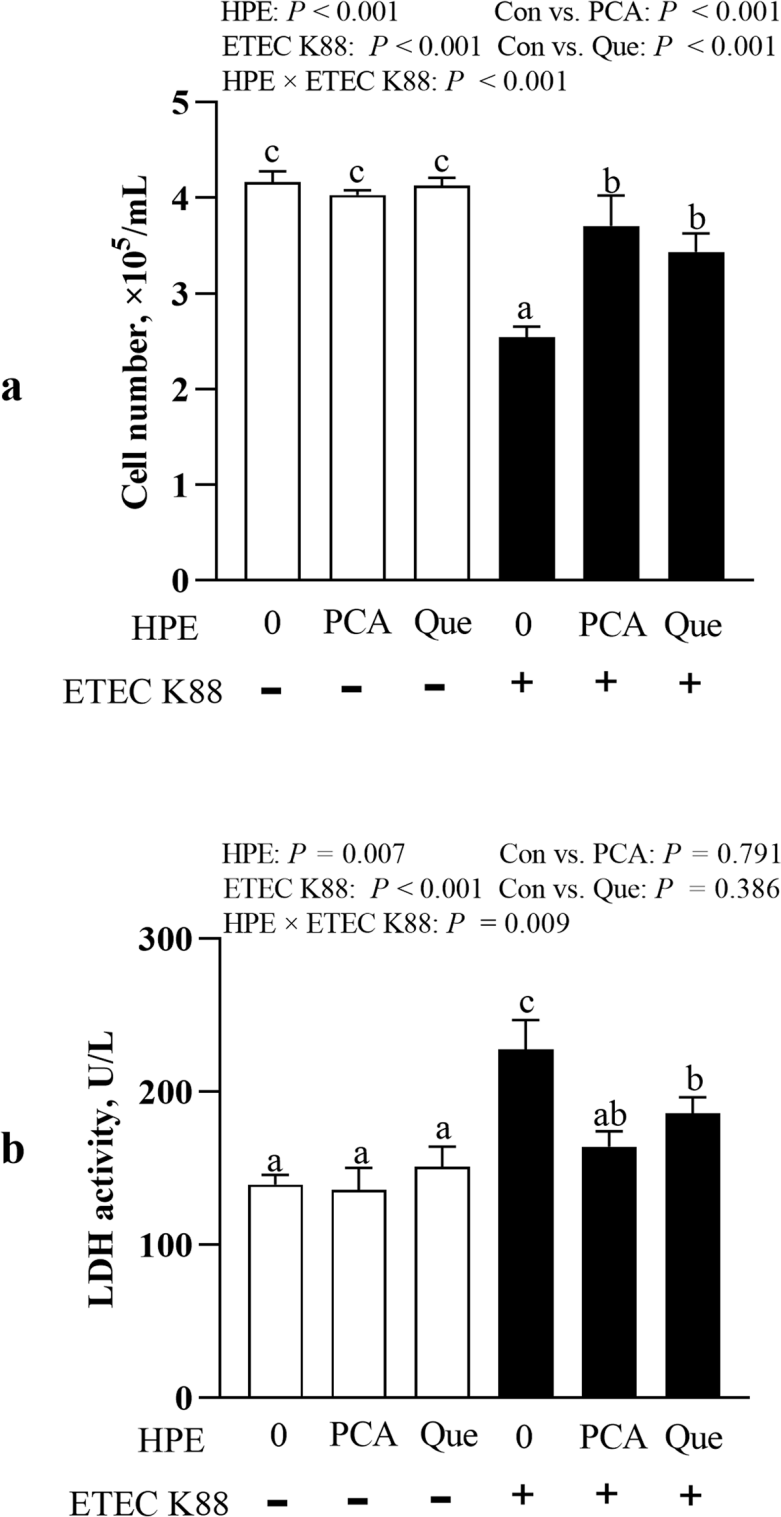
Effects of PCA and Que on cell number and LDH activity after infection with ETEC K88 in IPEC-1 cells. Cells were pretreated with 40 μmol/L PCA or 10 μmol/L Que for 24 h and then infected with or without 1 × 10^8^ ETEC K88/mL for 2 h. **a** Cell number. **b** LDH activity. Values are means ± SE, *n* = 6. ^a–c^Different letters represent a significant difference, *P <* 0.05. LDH, lactate dehydrogenases; IPEC-1, intestinal porcine epithelial cell 1

ETEC K88 infection reduced cell number and increased LDH activity (*P* < 0.001) in supernatant at 2 h (Fig. 2). HPE × ETEC K88 interactions were found for cell number (*P* < 0.001) and LDH activity (*P* = 0.009) in which pretreating with PCA or Que increased cell number and decreased LDH activity in ETEC-infected cells, however, there was no difference in cell number and LDH activity of non-ETEC K88-infected cells.

### PCA and Que protect epithelial cell barrier integrity after infection with ETEC K88 in IPEC-1 cells

**Fig. 3:**
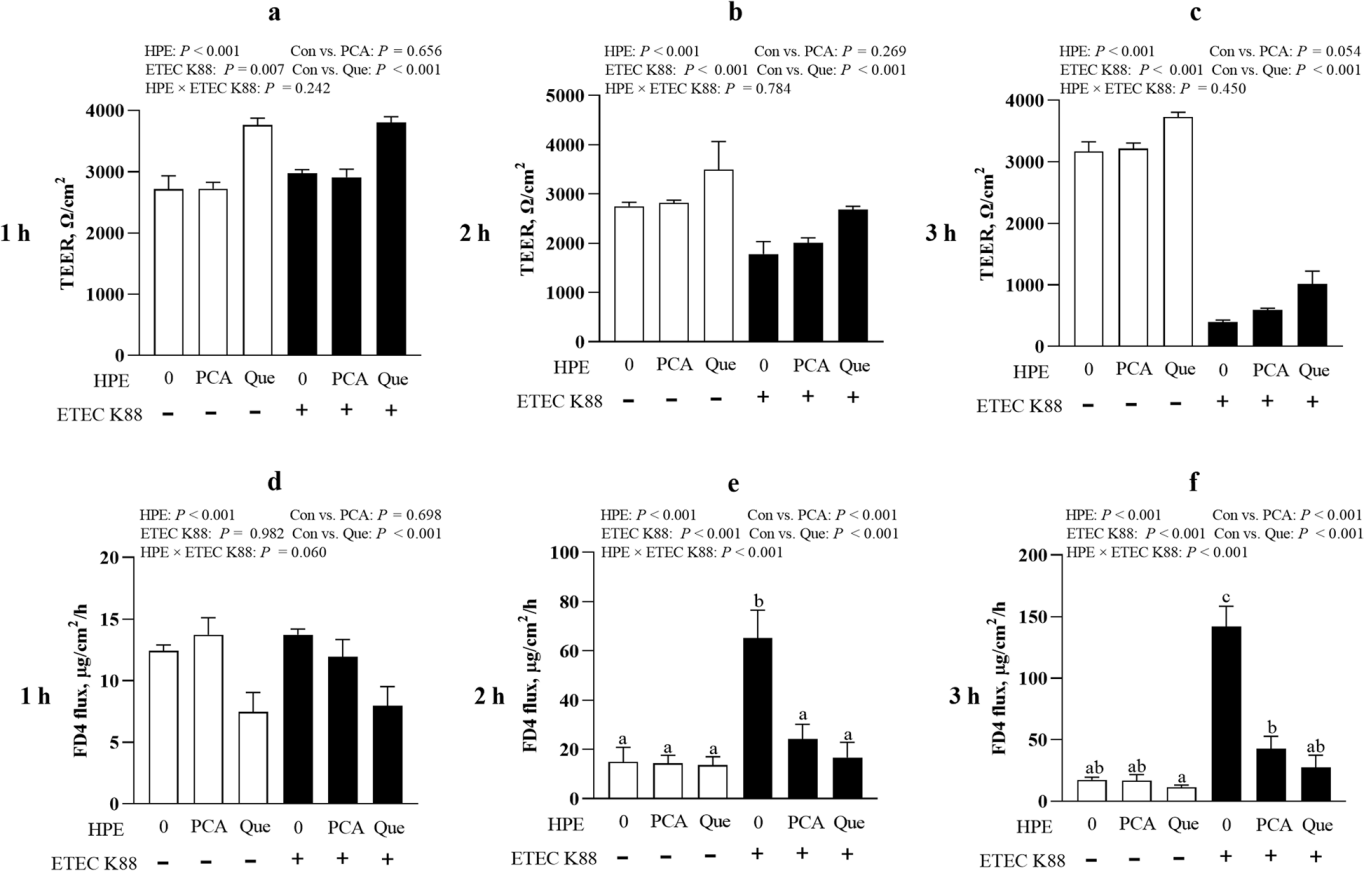
Effects of PCA and Que on TEER and FD4 permeability after infection with ETEC K88 in IPEC-1 cells. Cells were cultured with or without 40 μmol/L PCA or 10 μmol/L Que for 24 h, followed by infected with or without 1 × 10^8^ ETEC K88/mL for 1 h, 2 h and 3 h. **a**–**c** TEER. **d**–**f** FD4 permeability. Values are means ± SE, *n* = 6. ^a–c^Different letters represent a significant difference, *P <* 0.05. IPEC-1, intestinal porcine epithelial cell 1; TEER, transepithelial electrical resistance; FD4, fluorescein isothiocyanate (FITC)-labeled dextran 4 kDa

ETEC K88 infection reduced TEER value (*P* < 0.001) of IPEC-1 cell at 2 h and 3 h post stimulation (Fig. 3a-c). Pretreating with Que increased TEER at 1 h, 2 h and 3 h and pretreating with PCA did not influence TEER at 1 h and 2 h. There was a trend observed for TEER when pretreating with PCA at 3 h. No HPE × ETEC K88 interaction was found for TEER at 1 h, 2 h and 3 h in which Que pretreating improved TEER (*P* < 0.001) both in non-ETEC K88-treated groups or ETEC K88-treated groups.

ETEC K88 infection also raised FD4 permeability (*P* < 0.001) from cell apical membrane to basal membrane at 2 h and 3 h (Fig. 3d-f). A HPE × ETEC K88 interaction was found for FD4 permeability at 2 h and 3 h (*P* < 0.05) in which pretreating with PCA or Que decreased FD4 permeability (*P* < 0.05) compared with control cells in ETEC K88-treated groups, however, there was no difference for FD4 permeability at 2 h and 3 h among non-ETEC K88-treated groups.

### PCA and Que enhance tight junction protein expression and rescue distribution of tight junction proteins after infection with ETEC K88 in IPEC-1 cells

**Fig. 4:**
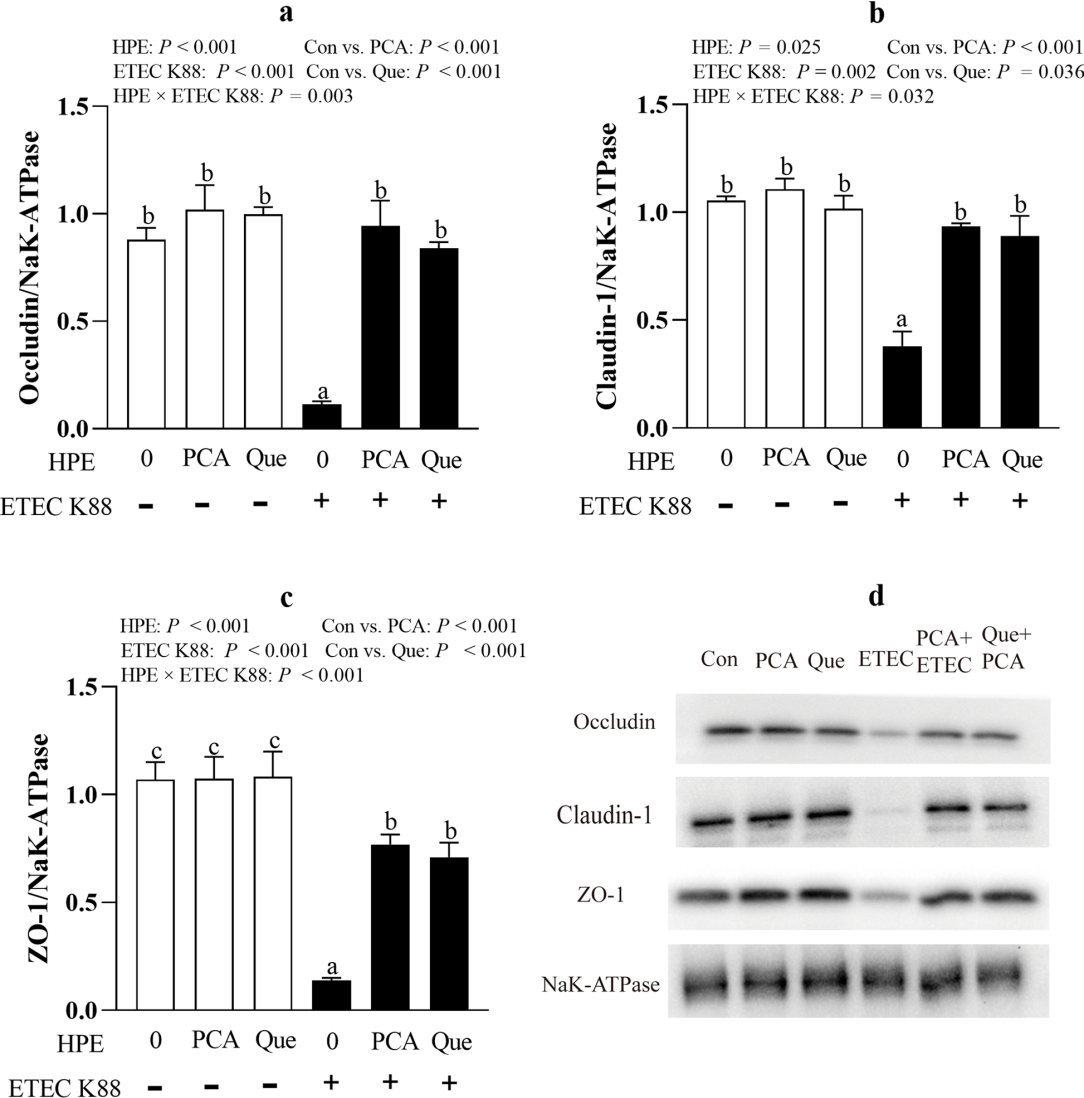
Effects of PCA and Que on tight junction protein expression after infection with ETEC K88 in IPEC-1 cells. Cells were cultured with or without 40 μmol/L PCA or 10 μmol/L Que for 24 h, and then infected with or without 1 × 10^8^ ETEC K88/mL for 2 h. **a**–**c** Tight junction protein expression. **d** Representative bands of tight junction proteins. Values are means ± SE, *n* = 6. ^a–c^Different letters represent a significant difference, *P* < 0.05. IPEC-1, intestinal porcine epithelial cell 1; ZO-1, zonula occludens-1

ETEC K88 infection reduced protein abundance of occludin (*P* < 0.001), claudin-1 (*P* < 0.05) and ZO-1 (*P* < 0.001) compared with control group (Fig. 4a–c). HPE × ETEC K88 interactions (*P* < 0.05) were found for protein abundance of occludin, claudin-1 and ZO-1 in which pretreating with PCA or Que enhanced protein abundance of these three tight junctions in ETEC-infected groups, whereas the protein abundance of occludin, claudin-1 and ZO-1 did not change among non-ETEC K88-infected groups.

**Fig. 5:**
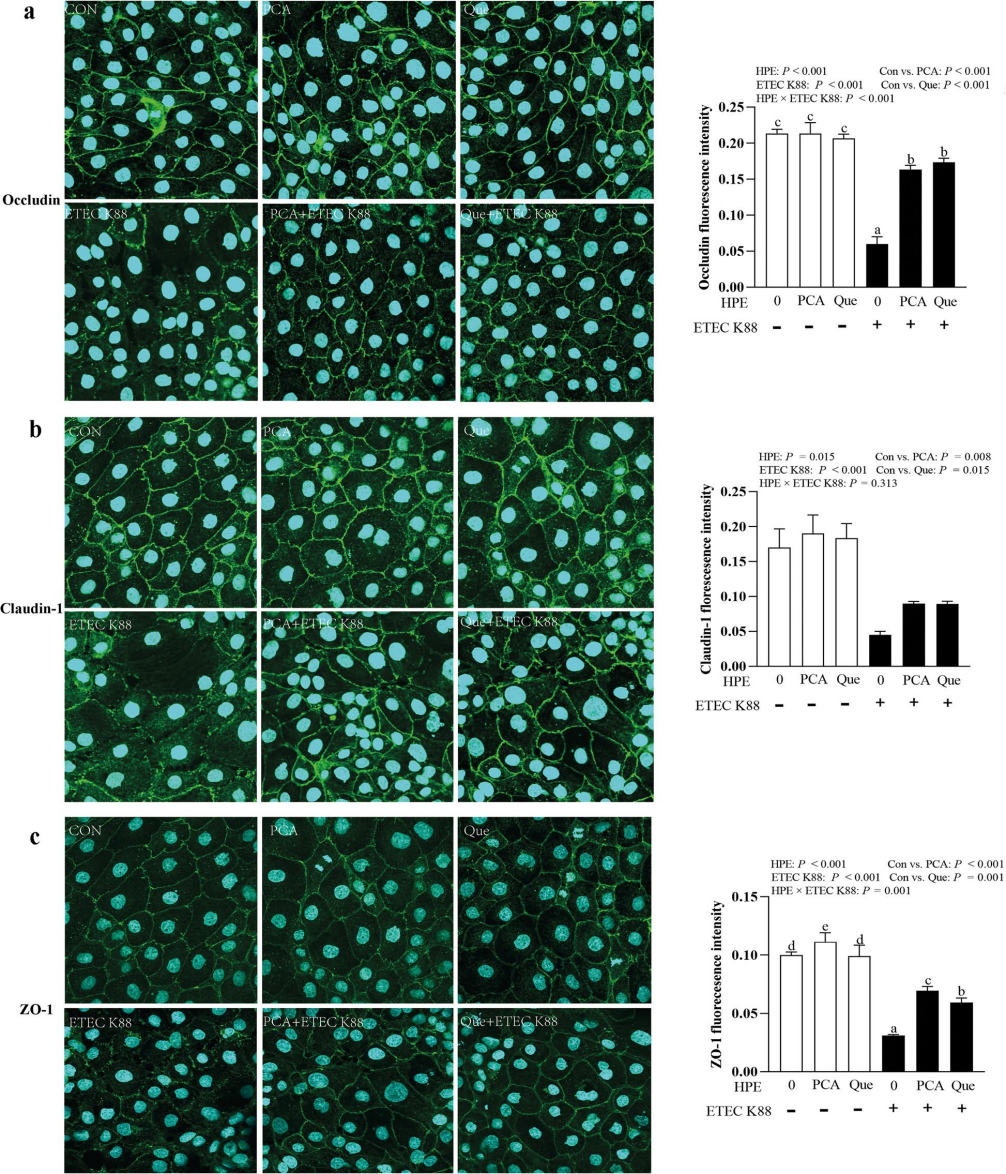
Effects of PCA and Que on tight junction protein distribution after infection with ETEC K88 in IPEC-1 cells. Cells were cultured in confocal dish with or without 40 μmol/L PCA or 10 μmol/L Que for 24 h, and then infected with or without 1 × 10^8^ ETEC K88/mL for 2 h. **a**–**c** Tight junction protein distribution. The spread and distribution of tight junction proteins were observed through a confocal microscope. Values are means ± SE, *n* = 6. ^a–e^Different letters represent a significant difference, *P* < 0.05. IPEC-1, intestinal porcine epithelial cell 1; ZO-1, zonula occludens-1

ETEC K88 infection broke the distribution of occludin, claudin-1 and ZO-1 (*P* < 0.001) around epithelial cells. Pretreating with PCA and Que rescued the location of these three tight junction proteins (*P* < 0.05) at cellular membrane after ETEC K88 stimulation (Fig. 5a–c). HPE × ETEC K88 interactions were found for occludin (*P* < 0.001) and ZO-1 (*P* < 0.05) inflorescences in which pretreating with PCA or Que improved protein inflorescences of occludin and ZO-1 in ETEC-infected groups, whereas the protein inflorescences of occludin did not change among non-ETEC K88-infected groups. Pretreating with PCA alone increased the protein inflorescences of ZO-1. However, pretreating with Que alone did not influence the protein inflorescences of ZO-1. PCA had better effects than Que in inhibiting the decrease of ZO-1 protein in ETEC K88-infected cells. No HPE × ETEC K88 interaction was found for claudin-1 in which both PCA and Que improved protein inflorescence of claudin-1 (*P* < 0.05) both in non-ETEC K88-treated groups or ETEC K88-treated groups.

### PCA and Que prevent cell necrosis after infection with ETEC K88 in IPEC-1 cells

**Fig. 6:**
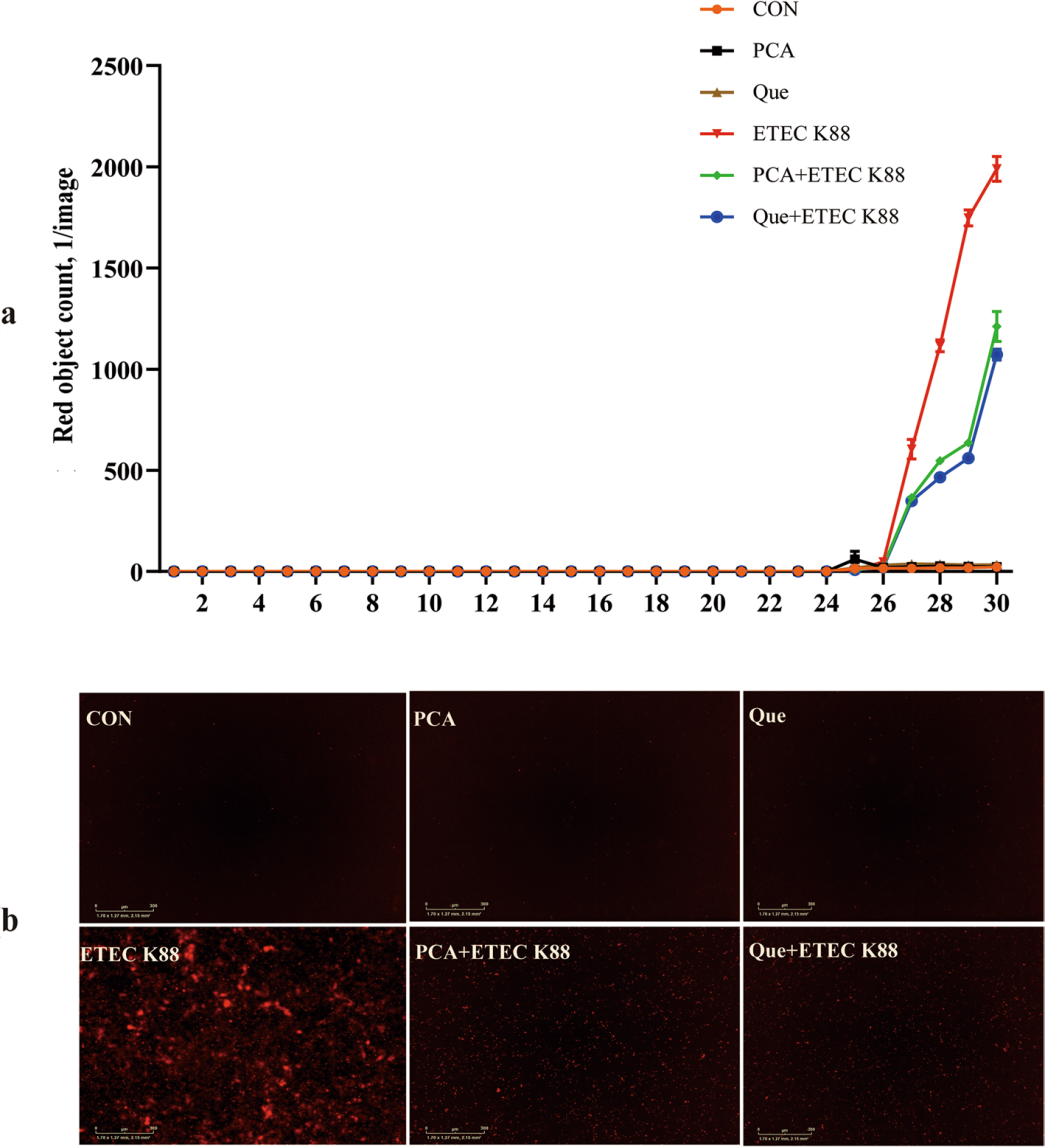
Effects of PCA and Que on cell necrosis after infection with ETEC K88 in IPEC-1 cells. Cells were cultured in CO_2_ incubator of IncuCyte ZOOM™ Live Cell Imaging System for 30 h. Firstly, cells were pre-treated with 40 μmol/L PCA or 10 μmol/L Que for 24 h and then infected with or without 1 × 10^8^ ETEC K88/mL for 6 h. **a** Dynamic observation of cell necrosis. **b** Representative images of cell necrosis at 6 h after ETEC K88 treatment (red dye was used to stain necrotic cells). IPEC-1, intestinal porcine epithelial cell 1

IncuCyte ZOOM™ Live Cell Imaging System was applied to detect cell necrosis in this experiment (Additional file 3). ETEC K88 infection increased cell necrosis ratio from 27 to 30 h compared with the control group (Fig. 6a). A HPE × ETEC K88 interaction was found at 28 and 30 h in which PCA or Que pretreating prevented the increase of cell necrosis in ETEC K88-infected groups, however, no difference was observed in non-ETEC K88-infected groups (Additional file 4). Images at 30 h after ETEC K88 infection also demonstrated that PCA or Que pretreating alleviated the increase of cell necrosis ratio after ETEC K88 stimulation (Fig. 6b).

### PCA and Que suppress pro-inflammatory cytokine secretion after infection with ETEC K88 in IPEC-1 cells

**Fig. 7:**
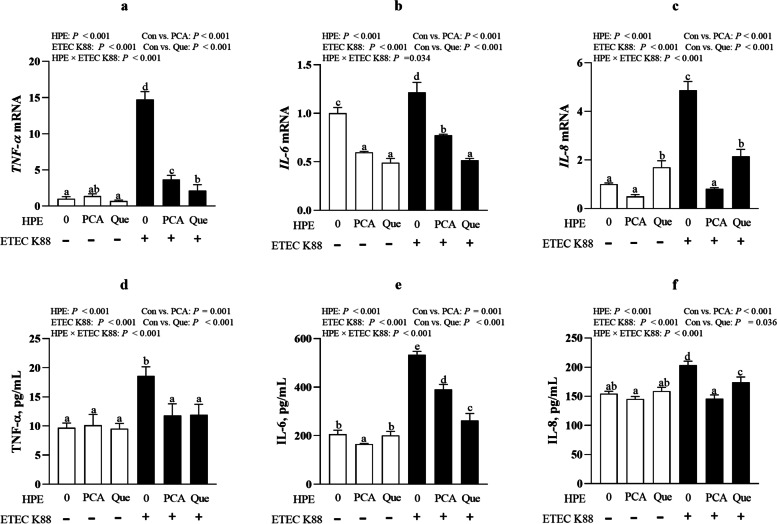
Effects of PCA and Que on proinflammatory cytokine secretion after infection with ETEC K88 in IPEC-1 cells. Cells were cultured with 40 μmol/L PCA or 10 μmol/L Que for 24 h, followed by infected with or without 1 × 10^8^ ETEC K88/mL for another 2 h. **a**–**c** mRNA expressions of *TNF-α, IL-8* and *IL-6*. **d**–**f** Protein concentration of TNF-α, IL-8 and IL-6. Values are means ± SE, *n* = 6. ^a–e^Different letters represent a significant difference, *P <* 0.05. IL-6, interleukin-6; IL-8, interleukin-8; TNF-α, tumor necrosis factor-α

ETEC K88 infection increased mRNA level of *TNF-α*, *IL-8* and *IL-6* (*P* < 0.001) (Fig. 7a–c). HPE × ETEC K88 interactions were found for *TNF-α*, *IL-8* and *IL-6* mRNA expression (*P* < 0.05) in which pretreating with PCA or Que down-regulated mRNA level of *TNF-α, IL-8* and *IL-6* (*P* < 0.05) among ETEC K88-infected cells. Moreover, Que had better effects than PCA in inhibiting *TNF-α* and *IL-6* mRNA expression in ETEC-infected cells. However, PCA had better effects than Que in inhibiting *IL-8* mRNA expression after ETEC K88 infection. Moreover, pretreating with PCA or Que alone decreased the *IL-6* mRNA expression in non-ETECK88-treated cells.

ETEC K88 infection also elevated secretion of TNF-α, IL-8 and IL-6 (*P* < 0.001) in IPEC-1 cells (Fig. 7d–f). HPE × ETEC K88 interactions (*P* < 0.001) were found for TNF-α, IL-8 and IL-6 protein contents in which PCA or Que suppressed the concentration of TNF-α, IL-8 and IL-6 (*P* < 0.05) in ETEC K88-infected groups, among which Que had better effect than PCA on inhibiting IL-6 secretion and PCA had better effect than Que on inhibiting IL-8 secretion. However, no difference of PCA and Que was observed for TNF-α concentration in non-ETEC K88-infected cells.

### PCA and Que inhibit TLR4 signaling pathway after infection with ETEC K88 in IPEC-1 cells

**Fig. 8:**
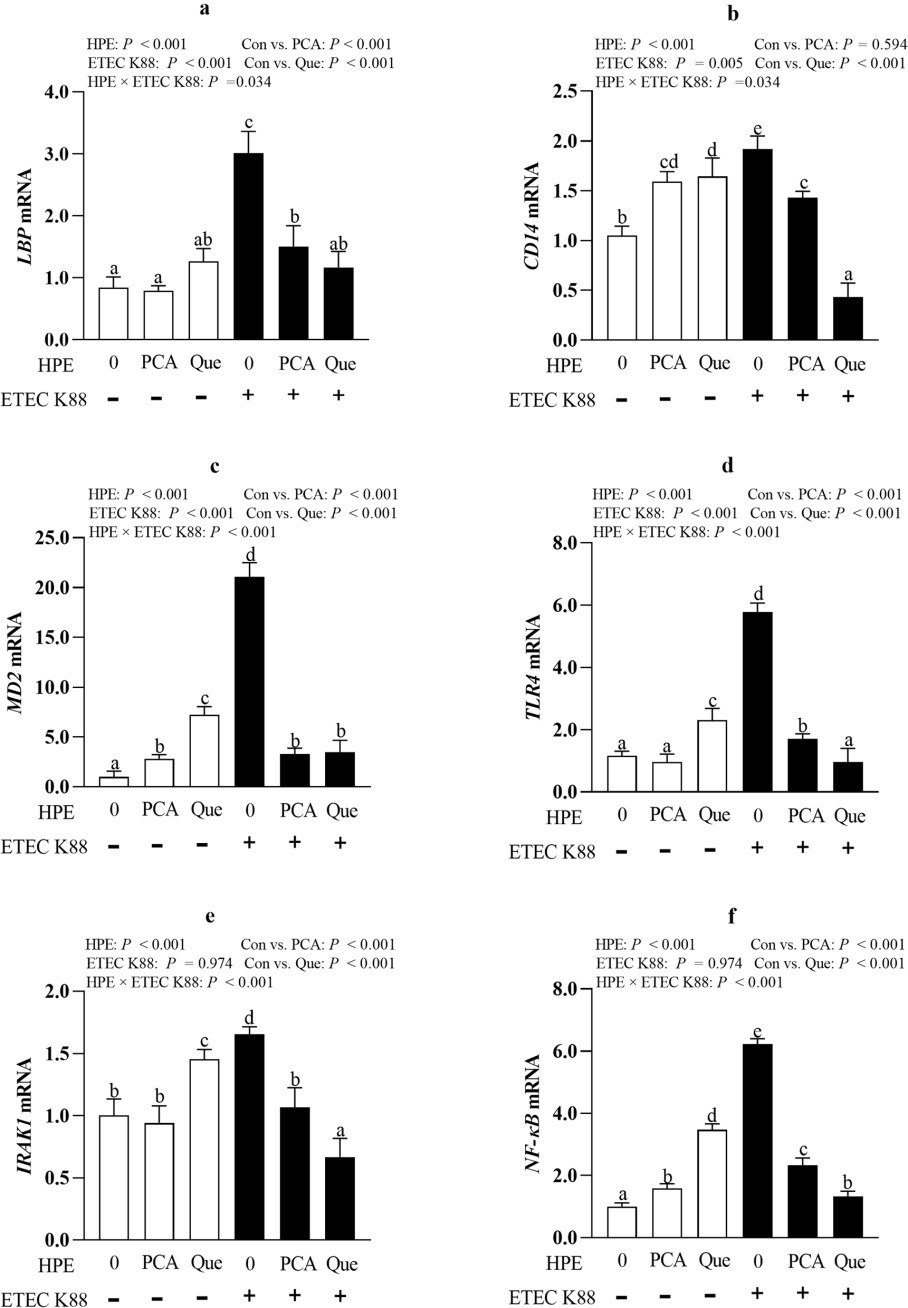
Effects of PCA and Que on mRNA abundance of TLR4 signaling pathway after infection with ETEC in IPEC-1 cells. Cells were pre-treated with 40 μmol/L PCA or 10 μmol/L Que for 24 h and then infected with or without 1 × 10^8^ ETEC K88/mL for 2 h. Values are means ± SE, *n* = 6. ^a–e^Different letters represent a significant difference, *P <* 0.05. IPEC-1, intestinal porcine epithelial cell 1. CD14, cluster differentiation factor-14; IRAK1, IL-1 receptor-associated kinase 1; LBP, LPS binding protein; MD2, myeloid differentiation factor-2; TLR4, toll-like receptor; NF-κB, nuclear factor-κB

ETEC K88 infection increased mRNA level of LPS binding protein (*LBP*), cluster differentiation factor-14 (*CD14*), myeloid differentiation factor-2 (*MD2*) and toll-like receptor 4 (*TLR4*) (*P* < 0.05) compared with control cells (Fig. 8a–f). HPE × ETEC K88 interactions were found for *LBP, CD14, MD2, TLR4,* IL-1 receptor-associated kinase 1 (*IRAK1*) and nuclear factor-κB (*NF-κB*) mRNA abundance in which Que pretreating decreased *LBP, CD14, MD2, TLR4, IRAK1* and *NF-κB* mRNA, and PCA decreased *LBP, MD2, TLR4, IRAK1* and *NF-κB* mRNA in ETEC K88-infected groups, however, Que increased *CD14, MD2, TLR4, IRAK1* and *NF-κB* mRNA expression and PCA increased *CD14, MD2* and *NF-κB* mRNA expression in non-ETEC K88-infected groups. Moreover, Que had better effects than PCA in inhibiting *CD14, TLR4, IRAK1* and *NF-κB* mRNA expression.

### PCA and Que suppress necroptosis signaling pathway after infection with ETEC K88 in IPEC-1 cells

**Fig. 9:**
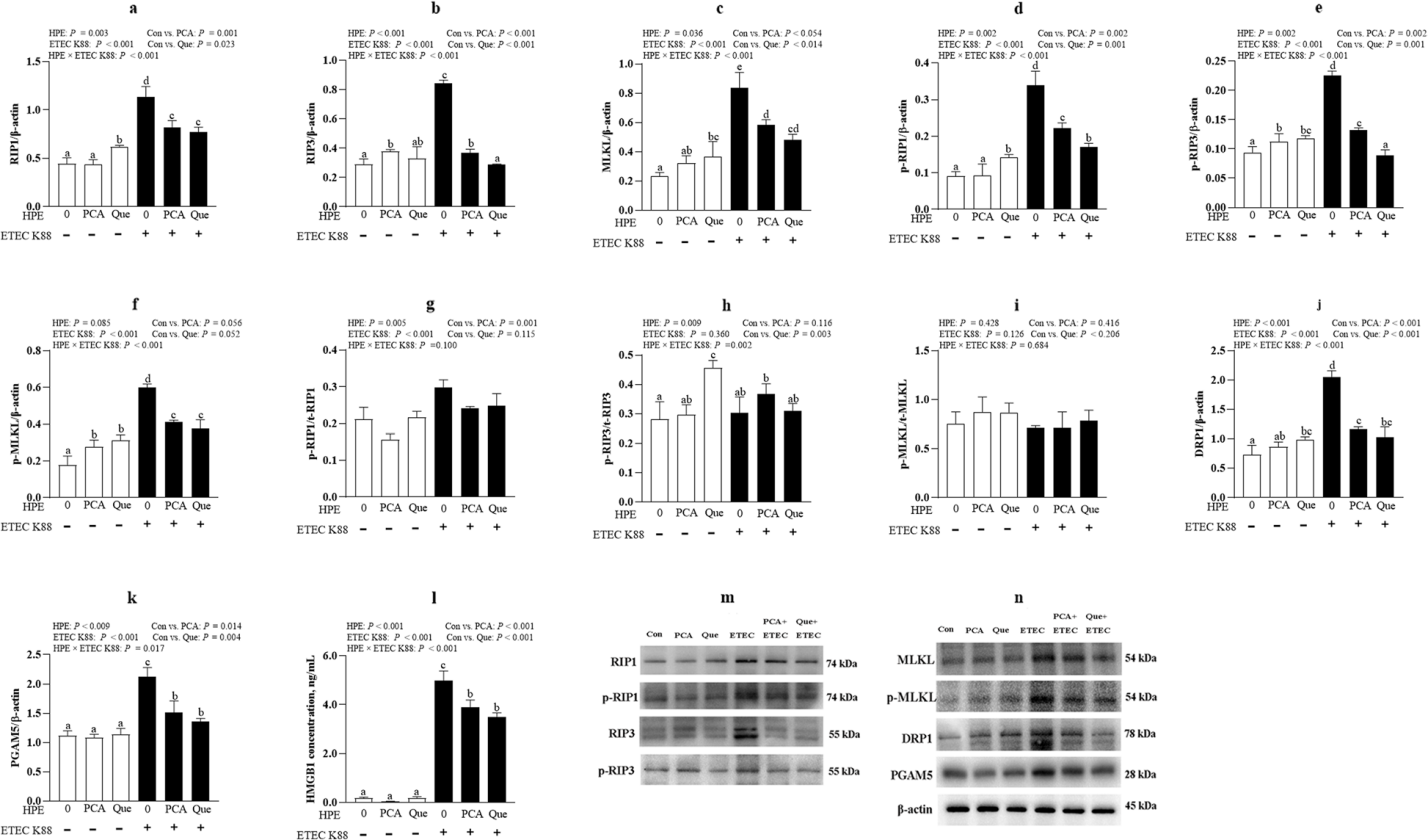
Effects of PCA and Que on protein expression of necroptosis signals after infection with ETEC K88 in IPEC-1 cells. Cells were pre-treated with 40 μmol/L PCA or 10 μmol/L Que for 24 h and then infected with or without 1 × 10^8^ ETEC K88/mL for 2 h. **a**–**k** Protein expression of t-RIP1, t-RIP3, t-MLKL, p-RIP1/t-RIP1, p-RIP3/t-RIP3, p-MLKL/t-MLKL, DRP1 and PGAM5. **l** Concentration of HMGB1. **m**–**n** Representative bands. Values are means ± SE, *n* = 6. ^a–e^Different letters represent a significant difference, *P <* 0.05. IPEC-1, intestinal porcine epithelial cell 1; t-RIP1, total receptor interacting protein 1; p-RIP1, phosphorylated-receptor interacting protein 1;t-RIP3, total receptor interacting protein 3; p-RIP3, phosphorylated-receptor interacting protein 3; t-MLKL, total mixed-lineage kinase domain-like protein; p-MLKL, phosphorylated-mixed-lineage kinase domain-like protein; PGAM5, phosphoglycerate mutase 5; DRP1, dynamin-related protein 1; HMGB1, high mobility group box 1

ETEC K88 infection up-regulated protein level of t-RIP1 (*P* < 0.001), t-RIP3 (*P* < 0.001), t-MLKL (*P* < 0.05), p-RIP1 (*P* < 0.001), p-RIP3 (*P* < 0.001), p-MLKL (*P* < 0.001), p-RIP1/t-RIP1 (*P* < 0.001), DRP1(*P* < 0.001), PGAM5 (*P* < 0.001) and HMGB1 (*P* < 0.001) compared with control group (Fig. 9a–n). HPE × ETEC K88 interactions were found for protein expression of t-RIP1 (*P* < 0.001), t-RIP3 (*P* < 0.001), t-MLKL (*P* < 0.001), p-RIP1 (*P* < 0.001), p-RIP3 (*P* < 0.001), p-MLKL (*P* < 0.001), p-RIP3/t-RIP3 (*P* < 0.05), DRP1 (*P* < 0.001), PGAM5 (*P* < 0.05) and HMGB1 (*P* < 0.001) in which PCA or Que pretreatment down-regulated protein abundance of t-RIP1, t-RIP3, t-MLKL, p-RIP1, p-RIP3, p-MLKL, DRP1, PGAM5 and HMGB1 in ETEC K88-infected groups, however, PCA increased the protein expression of t-RIP3, p-RIP3 and p-MLKL, and Que increased the protein expression of t-RIP1, t-MLKL, p-RIP1, p-RIP3, p-MLKL, p-RIP3/t-RIP3 and DRP1 in non-ETEC K88-infected groups.

### PCA and Que repress pyroptosis signaling pathway after infection with ETEC K88 in IPEC-1 cells

**Fig. 10:**
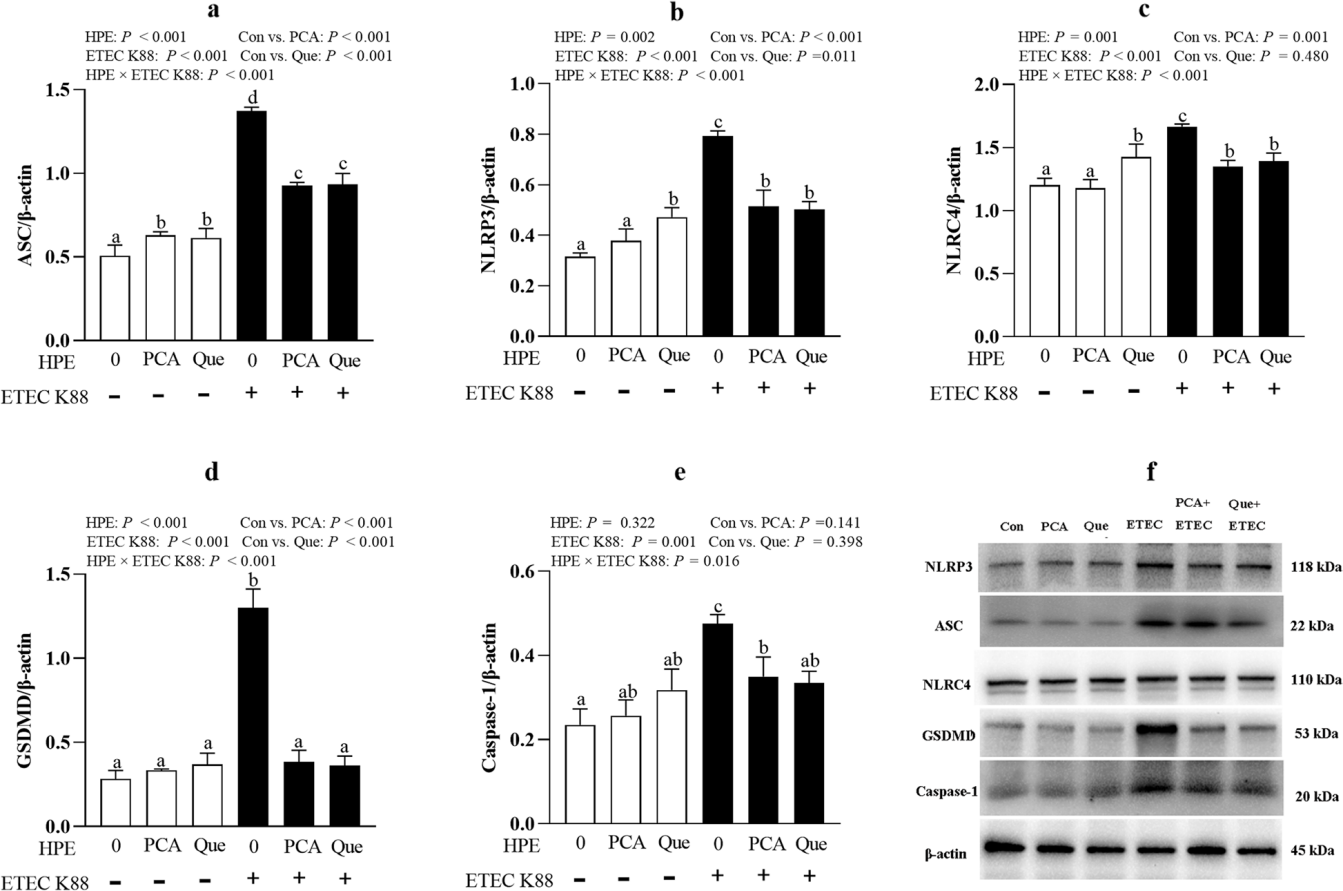
Effects of PCA and Que on protein expression of pyroptosis signals after infection with ETEC K88 in IPEC-1 cells. Cells were pre-treated with 40 μmol/L PCA or 10 μmol/L Que for 24 h and then infected with or without 1 × 10^8^ ETEC K88/mL for 2 h. **a**–**e** Protein concentration of pyroptosis signals. **f** Representative bands. Values are means ± SE, *n* = 6. ^a–d^Different letters represent a significant difference, *P <* 0.05. IPEC-1, intestinal porcine epithelial cell 1; NLRP3, nod-like receptor protein 3; NLRC4, nod-like receptors family CARD domain-containing protein; ASC, apoptosis-associated speck-like protein containing a CARD; GSDMD, gasdermin D

ETEC K88 infection increased protein abundance of NLRP3 (*P* < 0.001), ASC (*P* < 0.001), nod-like receptors family CARD domain-containing protein (NLRC4, *P* < 0.001), GSDMD (*P* < 0.001) and caspase-1 (*P* < 0.05) compared with control group (Fig. 10a–f). HPE × ETEC K88 interactions were found for ASC (*P* < 0.001), NLRP3 (*P* < 0.001), NLRC4 (*P* < 0.001), GSDMD (*P* < 0.001) and caspase-1 (*P* < 0.05) protein expression in which PCA or Que pretreatment down-regulated protein abundance of NLRP3, ASC, NLRC4, GSDMD and caspase-1 in ETEC K88-infected groups, whereas PCA increased the protein expression of ASC, and Que increased the protein expression of ASC, NLRP3 and NLRC4 in non- ETEC K88-infected groups.

## Discussion

ETEC is one of the most important pathogens causing diarrhea or sepsis in newborn livestock [25]. ETEC K88 is the most widely subtype, which can cause intestinal epithelial cell damage, inflammatory response, and even cell death. Studies have shown that ETEC can adhere to intestinal epithelial cells after entering the gut, then produce enterotoxin, causing intestinal inflammation and damage, which finally lead to endotoxemia or systemic infection [26]. So far, there is a lack of effective nutritional measures to alleviate ETEC infection.

Plant polyphenols are natural compounds widely existing in plants, and PCA and Que are the representative substances among plant polyphenols. It is previously reported that plant polyphenols were capable of inhibiting bacteria growth and thus protected gut health [27]. In the present study, PCA and Que did not inhibit ETEC growth. This is contrast with Chao and Yin [28] and Wang et al. [29] who found that PCA and Que had antibacterial effects against food spoilage bacteria including *Escherichia coli* O157:H7, *Staphylococcus aureus*, and *Bacillus cereus*. The discrepancies might be related to the difference in the concentration of plant polyphenols in our experiment. The contents of PCA and Que in their studies were 11.9 mg/g and 0.6 mg/g, respectively. However, the present study showed that PCA and Que decreased endotoxin secretion and bacterial adhesion after ETEC K88 infection. This is consistent with Gato et al. [30] who reported that corymbosum polyphenolic extract had anti-adhesive activity aginst *klebsiella pneumoniae*. However, little is known about the inhibitory effects of plant polyphenols on endotoxin secretion in vivo or in vitro. We demonstrated firstly that PCA and Que could inhibit ETEC adherence and endotoxin secretion. Since ETEC K88 needs firstly colonize to brush border of epithelial cells and then keeps releasing enterotoxins to induce water and electrolyte secretion, which indicates that pathogen-host cell contact is required for efficient toxin delivery [31]. The inhibition of the attachment of bacteria to epithelial cells has been a novel approach to prevent ETEC K88-induced diarrhoea in piglets [32]. We speculate that PCA and Que may inhibit bacterial adhesion to the epithelial cells and thus suppress endotoxin secretion.

Cell number reflects cell growth, and activity of LDH released into the supernatant from damaged cell reflects the degree of intestinal injury. In our study, expectedly, ETEC K88 infection decreased cell number and increased LDH activity, which suggested that ETEC K88 caused epithelial cell injury. Our data showed that PCA and Que increased cell number and decreased LDH activity, which indicated that PCA and Que protected IPEC-1 cell growth and mitigated cell damage. Similarly, investigation showed that pretreatment with PCA significantly reduced serum levels of LDH and malondialdehyde (MDA) after tert-butylhydroperoxide treatment in rat primary hepatocytes [33]. Que improved cell number and proliferation rate, and reduced the level of LDH of PC12 cells on the Alzheimer disease cell model induced by Aβ25–35 [34]. Que also increased cell viability and reduced LDH release in PC-12 cells when exposed to hydrogen peroxide [35, 36]. Therefore, in our present study, it is possible that PCA and Que prevent ETEC-induced cell number decrease and LDH increase by inhibiting bacterial adherence and endotoxin secretion.

The Intestinal barrier is composed of a layer of columnar epithelium and interepithelial tight junctions. Transepithelial electrical resistance (TEER) as a functional parameter for epithelial tightness [37], and FD4 permeability from mucosa to serosa as a functional parameter for epithelium and tight junctions are widely accepted in assessing barrier integrity in vitro [38]. Healthy barrier function possesses high TEER and low FD4 permeability [38]. In accordance with our previous study [23], ETEC infection reduced intestinal epithelial barrier function indicated by lower TEER and higher FD4 permeability. However, PCA and Que improved TEER value and reduced FD4 permeability after ETEC infection. Similarly, PCA and Que have been reported to increase TEER and decrease FD4 flux in several kinds of cells [36, 39, 40]. The tight junction proteins, includingoccludin, claudins, and ZO, are known as the most important organizers of the tight junctions [41]. In the current experiment, consistent with improved intestinal barrier function, PCA and Que rescued the protein expression and distribution of occludin, claudin-1 and ZO-1 as measured by confocal microscopy. In accordance with our study, PCA and Que have been reported to improve protein expression of occludin, claudin-1 and ZO-1 in vivo and vitro [42–44]. In our present study, it is possible that PCA and Que may partially protect intestinal barrier function via improving protein expression and rescuing distribution of intestinal tight junctions.

The death of epithelial cells can lead to impairment of barrier function and even intestinal damage [45]. Therefore, we next detected cell necrosis through IncuCyte ZOOM™ Live Cell Imaging System. In the current study, consistent with improved intestinal cell integrity, we found that PCA or Que supplementation decreased cell necrosis density. Although the beneficial effect of plant polyphenols on intestinal health has been studied, the research on PCA and Que regulating cell necrosis is very limited. Only reports from Chen et al. who reported that Que inhibited TNF-α induced HUVECs apoptosis in human umbilical vein endothelial cells (HUVECs) after TNF-α stimulation [46] and Kassab et al. who found PCA relived liver and kidney cell apoptosis in rats after monosodium glutamate intoxication [47]. These results indicate a novel and important role for plant polyphenols in inhibiting cell necrosis, which maybe ultimately contribute to improved barrier function.

ETEC is widely known to cause intestinal inflammation and excessive inflammation further exacerbates intestinal cell injury [2]. TLR4/ NF-κB signal pathway plays an important role in the development of inflammation and cell death, which has been intensively studied [48]. TLRs can be initiated by various factors include exogenous and endogenous substances, among which the most important factor is pathogens. Next, we explored whether PCA or Que could affect intestinal inflammation and relevant TLR4 signals. In the present study, PCA or Que suppressed mRNA and protein abundance of pro-inflammatory cytokines such as TNF-α, IL-8 and IL-6, which was associated with inhibiting TLR4 and its downstream signals such as *LBP*, *MD2, CD14, TLR4, IRAK1* and *NF-κB*, indicating a protective role in preventing intestinal inflammation via TLR4 signaling pathway. Currently, abundant research has been found that supplementation of plant polyphenols can modulate intestinal inflammation and cell death. For example, Tang et al. found that Que reduced LPS-induced increased of proinflammatory cytokines including TNF-α, IL-1β and IL-6 in RAW264.7 cells [49]. Chen et al. also found that Que inhibited TNF-α or IL-1β- induced inflammation in human umbilical vein endothelial cells or ARPE-19 Cells via downregulating NF-KB signaling pathway [46, 50, 51]. PCA has been reported to attenuate secretion of proinflammatory cytokines, including TNF-α, IL-1β, and IL-6 expression via efficiently inhibiting NF-ΚB activation after LPS challenge [52, 53]. Polyphenols including flavonoids, phenolic acids, phenolic alcohols, can reduce inflammation via modulation of the TLR4 signaling pathway [54]. In our current study, consistent with these previous studies, it is possible that the protective effects of PCA and Que on intestinal inflammation were associated with inhibiting TLR4/NF-ΚB signaling pathway.

Inflammation can reversely contribute to cell death. To elucidate the mechanism(s) by which PCA and Que prevent intestinal inflammation and injury, we next investigated whether necroptosis and pyroptosis signaling pathways were involved in ETEC-caused inflammation and cell necrosis. Necroptosis is a newly identified pathway of regulated necrosis, which is associated with many intestinal disorders [13]. Necroptosis is also regarded as a highly pro-inflammatory mode of cell death [16]. Various stimuli can lead to activation of cell necroptosis [55]. Initially, the intracellular adapter molecules FADD and TRADD recruit RIP1, which subsequently recruits RIP3 to assemble the necrosome including phosphorylated RIP1, RIP3 and MLKL [56]. In the present study, we found that PCA and Que downregulated ETEC K88-induced protein expression of t-RIP1, p-RIP1, t-RIP3, p-RIP3, p-MLKL, DRP1, PGAM5 and HMGB1. However, little research was carried out to investigate plant polyphenols on regulating necroptosis signals. Only Fan et al. reported that Que prevented necroptosis of oligodendrocytes in rats after spinal cord injury [57]. Recently, Liu et al. also found that Que alleviated cadmium-induced necroptosis in the chicken brain [58]. At present, there is no study exploring the role of PCA on necroptosis signal pathway. In our current study, we uncovered for the first time that similar to Que, PCA also exhibited protective effects in suppressing necroptosis signaling pathway. So, PCA or Que may regulate ETEC-caused cell inflammatory response and damage associated with inhibition of necroptosis signaling pathway.

Pyroptosis is caspase-1–dependent cell death, which is morphologically and mechanistically distinct from other forms of cell death [56]. Pyroptosis is inherently inflammatory, and is triggered by various pathological stimuli and crucial for controlling microbial infections [59]. After stimulation, membrane nod-like receptors, including NLRP3, recruits the adapter protein ASC or directly recruits caspase-1 to be leavaged. NLRC4 can directly interact with caspase-1 when overexpressed [60]. Pyroptosis is found to be involved in dysfunction of intestinal injury [61]. In the present study, consistent with necroptosis, we found that PCA or Que decreased protein level of NLRP3, ASC, NLRC4, caspase-1 and IL-18 after ETEC K88 infection, suggesting a protective role on inhibiting pyroptosis signaling pathway activation. Until now, there is little research about effect of plant polyphenols especially PCA or Que on pyroptosis signals. Only Liu et al. reported that apple polyphenols extract ameliorated dextran sulfate sodium-induced acute ulcerative colitis through inhibiting intestinal epithelial cell apoptosis and pyroptosis pathway [62]. Luo et al. reported that Que possessed a protective effect on macrophages pyroptosis via TLR2/Myd88/NF-ΚB pathway [63]. Until now, there was no other report about the effect of PCA on pyroptosis signaling pathway. We showed for the first time that PCA could suppress cell pyroptosis signals to alleviate intestinal inflammation and injury. It is possible that the beneficial role of PCA and Que on intestinal damage and inflammation was associated with suppressing necroptosis and pyroptosis signaling pathways, which suggesting a promising role of plant polyphenols in protecting gut health.

## Conclusions

In summary, plant polyphenols including PCA and Que play a beneficial role in protecting against ETEC K88-caused intestinal inflammation, cell damage and barrier impairment. It is possible that the beneficial role of PCA and Que on intestinal cells are associated with inhibition of necroptosis and pyroptosis signaling pathways. Targeting necroptosis and pyroptosis by plant polyphenols, especially PCA and Que may open a new therapeutic window for treating gastrointestinal diseases.

## Supplementary Information

**Additional file 1.** Primers used for real-time PCR analyses.

**Additional file 2.** Antibacterial effects of PCA and Que on ETEC K88 growth. After inoculating ETEC K88 into LB medium, 40 μmol/L PCA or 10 μmol/L Que or PBS were added into the holes to incubate for 24 h at 37 °C.

**Additional file 3.** Real time dynamic analysis of necrosis in IPEC-1 cells for control group. Necrotic cells were dyed with red yoyo-3 and monitored from 0 to 30 h. Real time dynamic analysis of necrosis in IPEC-1 cells for PCA group. Necrotic cells were dyed with red yoyo-3 and monitored from 0 to 30 h. Real time dynamic analysis of necrosis in IPEC-1 cells for Que group. Necrotic cells were dyed with red yoyo-3 and monitored from 0 to 30 h. Real time dynamic analysis of necrosis in IPEC-1 cells for ETEC K88 group. Necrotic cells were dyed with red yoyo-3 and monitored from 0 to 30 h. Real time dynamic analysis of necrosis in IPEC-1 cells for PCA+ ETEC K88 group. Necrotic cells were dyed with red yoyo-3 and monitored from 0 to 30 h. Real time dynamic analysis of cell necrosis in IPEC-1 cells for Que + ETEC K88 group. Necrotic cells were dyed with red yoyo-3 and monitored from 0 to 30 h.

**Additional file 4. **Effects of PCA and Que on cell necrosis in IPEC-1 cells infected with ETEC K88 at 28 and 30 h (4 and 6 h after ETEC K88 infection, respectively). Cells were pre-treated with 40 μmol/L PCA or 10 μmol/L Que for 24 h and then infected with or without 1 × 10^8^ ETEC K88/mL for 6 h. Values are means ± SE, *n* = 6. ^a–c^Different letters represent a significant difference, *P <* 0.05. IPEC-1, intestinal porcine epithelial cell 1.

## Data Availability

The data used to support the findings of this study are available from the corresponding author upon reasonable request.

## Abbreviations

ASC: Apoptosis-associated speck-like protein containing a CARD
CD14: Cluster differentiation factor-14
DRP1: Dynamin-related protein 1
ETEC: Enterotoxigenic *Escherichia coli*
FADD: Fas-associated death domain protein
FD4: Fluorescein isothiocyanate-labeled dextran 4KD
GSDMD: Gasdermin D
HMGB1: High mobility group box 1
IPEC: Intestinal porcine epithelial cells
IL-6: Interleukin-6
IL-8: Interleukin-8
IRAK1: IL-1 receptor-associated kinase 1
LBP: LPS binding protein
LDH: Lactate dehydrogenases
NF-κB: Nuclear factor-κB
NLRC4: Nod-like receptors family CARD domain-containing protein 4
NLRP3: Nod-like receptor protein 3
MD2: Myeloid differentiation factor-2
MLKL: Mixed lineage kinase domain-like protein
PCA: Protocatechuic acid
PGAM5: Phosphoglycerate mutase 5
RIP1: Receptor interacting protein kinase 1
TEER: Transepithelial electrical resistance
TLR4: Toll-like receptors 4
TNF-α: Tumor necrosis factor-α

## References

[1] Khalil IA, Troeger C, Blacker BF, Rao PC, Brown A, Atherly DE (2018). Morbidity and mortality due to shigella and enterotoxigenic *Escherichia coli* diarrhoea: the Global Burden of Disease Study 1990-2016. Lancet Infect Dis.

[2] Brubaker J, Zhang XY, Bourgeois AL, Harro C, Sack DA, Chakraborty S. Intestinal and systemic inflammation induced by symptomatic and asymptomatic enterotoxigenic *E. coli* infection and impact on intestinal colonization and ETEC specific immune responses in an experimental human challenge model. Gut Microbes. 2021;13(1). 10.1080/19490976.2021.1891852. 10.1080/19490976.2021.1891852 PMC7919917 33645430

[3] Sun YW, Kim SW (2017). Intestinal challenge with enterotoxigenic *Escherichia coli* in pigs, and nutritional intervention to prevent postweaning diarrhea. Anim Nutr.

[4] Efenberger-Szmechtyk M, Nowak A, Czyzowska A (2021). Plant extracts rich in polyphenols: antibacterial agents and natural preservatives for meat and meat products. Crit Rev Food Sci..

[5] Golabek A, Kowalska K, Olejnik A. Polyphenols as a diet therapy concept for endometriosis-current opinion and future perspectives. Nutrients. 2021;13(4). 10.3390/nu13041347. 10.3390/nu13041347 PMC8074087 33919512

[6] Zhang S, Gai Z, Gui T, Chen J, Chen Q, Li Y. Antioxidant effects of protocatechuic acid and protocatechuic aldehyde: old wine in a new bottle. Evid Based Co Alt. 2021. 10.1155/2021/6139308. 10.1155/2021/6139308 PMC8592717 34790246

[7] Xu B, Qin W, Xu Y, Yang W, Chen Y, Huang J, et al. Dietary quercetin supplementation attenuates diarrhea and intestinal damage by regulating gut microbiota in weanling piglets. Oxidative Med Cell Longev. 2021. 10.1155/2021/6221012. 10.1155/2021/6221012 PMC8689231 34950418

[8] Han XJ, Xu TS, Fang QJ, Zhang HJ, Yue LJ, Hu G, et al. Quercetin hinders microglial activation to alleviate neurotoxicity via the interplay between NLRP3 inflammasome and mitophagy. Redox Biol. 2021:44. 10.1016/j.redox.2021.102010. 10.1016/j.redox.2021.102010 PMC8182123 34082381

[9] Porras D, Nistal E, Martinez-Florez S, Pisonero-Vaquero S, Olcoz JL, Jover R (2017). Protective effect of quercetin on high-fat diet-induced non-alcoholic fatty liver disease in mice is mediated by modulating intestinal microbiota imbalance and related gut-liver axis activation. Free Radical Bio Med.

[10] Zou Y, Wei HK, Xiang QH, Wang J, Zhou YF, Peng J (2016). Protective effect of quercetin on pig intestinal integrity after transport stress is associated with regulation oxidative status and inflammation. J Vet Med Sci.

[11] Wang YB, Wang YY, Wang BK, Mei XQ, Jiang SQ, Li WF (2019). Protocatechuic acid improved growth performance, meat quality, and intestinal health of Chinese yellow-feathered broilers. Poultry Sci.

[12] Xu X, Wei Y, Hua H, Jing X, Zhu H, Xiao K, et al. Polyphenols sourced from Ilex latifolia thunb. relieve intestinal injury via modulating ferroptosis in weanling piglets under oxidative stress. Antioxidants. 2022;11(5). 10.3390/antiox11050966. 10.3390/antiox11050966 PMC9137833 35624829

[13] Weinlich R, Oberst A, Beere HM, Green DR (2017). Necroptosis in development, inflammation and disease. Nat Rev Mol Cell Bio.

[14] Kovacs SB, Miao EA (2017). Gasdermins: effectors of pyroptosis. Trends Cell Biol.

[15] Tang R, Xu J, Zhang B, Liu J, Liang C, Hua J, et al. Ferroptosis, necroptosis, and pyroptosis in anticancer immunity. J Hematol Oncol. 2020;13(1). 10.1186/s13045-020-00946-7. 10.1186/s13045-020-00946-7 PMC7418434 32778143

[16] Pasparakis M, Vandenabeele P (2015). Necroptosis and its role in inflammation. Nature..

[17] Yu P, Zhang X, Liu N, Tang L, Peng C, Chen X. Pyroptosis: mechanisms and diseases. Signal Transduct Tar. 2021;6(1). 10.1038/s41392-021-00507-5. 10.1038/s41392-021-00507-5 PMC8005494 33776057

[18] Yuan YY, Xie KX, Wang SL, Yuan LW (2018). Inflammatory caspase-related pyroptosis: mechanism, regulation and therapeutic potential for inflammatory bowel disease. Gastroenterol Rep.

[19] Fritsch M, Gunther SD, Schwarzer R, Albert MC, Schorn F, Werthenbach JP (2019). Caspase-8 is the molecular switch for apoptosis, necroptosis and pyroptosis. Nature..

[20] Schwarzer R, Jiao HP, Wachsmuth L, Tresch A, Pasparakis M (2020). FADD and caspase-8 regulate gut homeostasis and inflammation by controlling MLKL- and GSDMD-mediated death of intestinal epithelial cells. Immunity..

[21] Liu YL, Xu Q, Wang Y, Liang TZ, Li XG, Wang D, et al. Necroptosis is active and contributes to intestinal injury in a piglet model with lipopolysaccharide challenge. Cell Death Dis. 2021;12(1). 10.1038/s41419-020-03365-1. 10.1038/s41419-020-03365-1 PMC7801412 33431831

[22] Xiao K, Liu CC, Qin Q, Zhang Y, Wang XY, Zhang J (2020). EPA and DHA attenuate deoxynivalenol-induced intestinal porcine epithelial cell injury and protect barrier function integrity by inhibiting necroptosis signaling pathway. FASEB J.

[23] Xiao K, Yang Y, Zhang Y, Lv Q, Huang F, Wang D, et al. Long chain PUFA ameliorate ETEC-induced intestinal inflammation and cell injury by modulating pyroptosis and necroptosis signaling pathways in IPEC-1 cells. Br J Nutr 2022:1–36. doi: 10.1017/S0007114521005092. 10.1017/S0007114521005213 35470787

[24] Brugger SD, Baumberger C, Jost M, Jenni W, Brugger U, Mühlemann K (2012). Automated counting of bacterial colony forming units on agar plates. PLoS One.

[25] Laird TJ, Abraham S, Jordan D, Pluske JR, Hampson DJ, Trott DJ, et al. Porcine enterotoxigenic *Escherichia coli*: Antimicrobial resistance and development of microbial-based alternative control strategies. Vet Microbiol. 2021:258. 10.1016/j.vetmic.2021.109117. 10.1016/j.vetmic.2021.109117 34049073

[26] Dubreuil JD, Isaacson RE, Schifferli DM (2016). Animal enterotoxigenic *Escherichia coli*. EcoSal Plus.

[27] Wan MLY, Co VA, El-Nezami H (2021). Dietary polyphenol impact on gut health and microbiota. Crit Rev Food Sci.

[28] Chao CY, Yin MC (2009). Antibacterial effects of roselle calyx extracts and protocatechuic acid in ground beef and apple juice. Foodborne Pathog Dis.

[29] Wang SN, Yao JY, Zhou B, Yang JX, Chaudry MT, Wang M (2018). Bacteriostatic effect of quercetin as an antibiotic alternative in vivo and its antibacterial mechanism in vitro. J Food Protect.

[30] Gato E, Rosalowska A, Martinez-Guitian M, Lores M, Bou G, Perez A. Anti-adhesive activity of a Vaccinium corymbosum polyphenolic extract targeting intestinal colonization by Klebsiella pneumoniae. Biomed Pharmacother. 2020:132. 10.1016/j.biopha.2020.110885. 10.1016/j.biopha.2020.110885 33113420

[31] Kumar P, Kuhlmann FM, Bhullar K, Yang H, Vallance BA, Xia L, Fleckenstein JM (2016). Dynamic interactions of a conserved enterotoxigenic *Escherichia coli* adhesin with intestinal mucins govern epithelium engagement and toxin delivery. Infect Immun.

[32] González-Ortiz G, Pérez JF, Hermes RG, Molist F, Jiménez-Díaz R, Martín-Orúe SM (2014). Screening the ability of natural feed ingredients to interfere with the adherence of enterotoxigenic *Escherichia coli* (ETEC) K88 to the porcine intestinal mucus. Brit J Nutr.

[33] Liu CL, Wang JM, Chu CY, Cheng MT, Tseng TH (2002). In vivo protective effect of protocatechuic acid on tert-butyl hydroperoxide-induced rat hepatotoxicity. Food Chem Toxicol.

[34] Yu X, Li Y, Mu X (2020). Effect of quercetin on PC12 Alzheimer's disease cell model induced by abeta 25-35 and its mechanism based on sirtuin1/Nrf2/HO-1 pathway. Biomed Res Int.

[35] Bao DK, Wang JK, Pang XB, Liu HL. Protective effect of quercetin against oxidative stress-induced cytotoxicity in rat pheochromocytoma (PC-12) cells. Molecules. 2017;22(7). 10.3390/molecules22071122. 10.3390/molecules22071122 PMC6152301 28684704

[36] Li Y, Zhou S, Li J, Sun Y, Hasimu H, Liu R (2015). Quercetin protects human brain microvascular endothelial cells from fibrillar beta-amyloid1-40-induced toxicity. Acta Pharm Sin B.

[37] Petto C, Lesko S, Gäbel G, Böttner M, Wedel T, Kacza J, Pfannkuche H (2011). Establishment and characterization of porcine colonic epithelial cells grown in primary culture. Cells Tissues Organs.

[38] Blikslager AT, Moeser AJ, Gookin JL, Jones SL, Odle J (2007). Restoration of barrier function in injured intestinal mucosa. Physiol Rev.

[39] Fuentes J, Brunser O, Atala E, Herranz J, de Camargo AC, Zbinden-Foncea H, et al. Protection against indomethacin-induced loss of intestinal epithelial barrier function by a quercetin oxidation metabolite present in onion peel: In vitro and in vivo studies. J Nutr Biochem. 2022:100. 10.1016/j.jnutbio.2021.108886. 10.1016/j.jnutbio.2021.108886 34670110

[40] Song LQ, Wu T, Zhang L, Wan J, Ruan Z. Chlorogenic acid improves the intestinal barrier by relieving endoplasmic reticulum stress and inhibiting ROCK/MLCK signaling pathways. Food Funct. 2022. 10.1039/d1fo02662c. 10.1039/d1fo02662c 35353100

[41] Suzuki T (2013). Regulation of intestinal epithelial permeability by tight junctions. Cell Mol Life Sci.

[42] Yao X, Mei Y, Mao W (2021). Quercetin improves mitochondrial function and inflammation in H_2_O_2_-induced oxidative stress damage in the gastric mucosal epithelial cell by regulating the PI3K/AKT signaling pathway. Evid Based Complement Altern Med.

[43] Chuenkitiyanon S, Pengsuparp T, Jianmongkol S (2010). Protective effect of quercetin on hydrogen peroxide-induced tight junction disruption. Int J Toxicol.

[44] Hu RZ, He ZY, Liu M, Tan JJ, Zhang HF, Hou DX, et al. Dietary protocatechuic acid ameliorates inflammation and up-regulates intestinal tight junction proteins by modulating gut microbiota in LPS-challenged piglets. J Anim Sci Biotechno. 2020;11(1). 10.1186/s40104-020-00492-9. 10.1186/s40104-020-00492-9 PMC7487840 32944233

[45] Williams JM, Duckworth CA, Burkitt MD, Watson AJM, Campbell BJ, Pritchard DM (2015). Epithelial cell shedding and barrier function: a matter of life and death at the small intestinal villus tip. Vet Pathol.

[46] Chen T, Zhang X, Zhu G, Liu H, Chen J, Wang Y (2020). Quercetin inhibits TNF-alpha induced HUVECs apoptosis and inflammation via downregulating NF-kB and AP-1 signaling pathway in vitro. Medicine (Baltimore).

[47] Kassab RB, Theyab A, Al-Ghamdy AO, Algahtani M, Mufti AH, Alsharif KF (2022). Protocatechuic acid abrogates oxidative insults, inflammation, and apoptosis in liver and kidney associated with monosodium glutamate intoxication in rats. Environ Sci Pollut R.

[48] Ben DF, Yu XY, Ji GY, Zheng DY, Lv KY, Ma B (2012). TLR4 mediates lung injury and inflammation in intestinal ischemia-reperfusion. J Surg Res.

[49] Tang J, Diao P, Shu XH, Li L, Xiong LD. Quercetin and quercitrin attenuates the inflammatory response and oxidative stress in LPS-induced RAW264.7 cells: in vitro assessment and a theoretical model. Biomed Res Int 2019;2019. doi: 10.1155/2019/7039802. 10.1155/2019/7039802 PMC6855062 31781635

[50] Cheng SC, Huang WC, Pang JHS, Wu YH, Cheng CY. Quercetin inhibits the production of IL-1beta-induced inflammatory cytokines and chemokines in ARPE-19 Cells via the MAPK and NF-kappaB signaling pathways. Int J Mol Sci. 2019;20(12). 10.3390/ijms20122957. 10.3390/ijms20122957 PMC6628093 31212975

[51] Le K, Song ZP, Deng J, Peng X, Zhang J, Wang L (2020). Quercetin alleviates neonatal hypoxic-ischemic brain injury by inhibiting microglia-derived oxidative stress and TLR4-mediated inflammation. Inflamm Res.

[52] Adedara IA, Fasina OB, Ayeni MF, Ajayi OM, Farombi EO (2019). Protocatechuic acid ameliorates neurobehavioral deficits via suppression of oxidative damage, inflammation, caspase-3 and acetylcholinesterase activities in diabetic rats. Food Chem Toxicol.

[53] Wei M, Chu X, Jiang L, Yang X, Cai Q, Zheng C (2012). Protocatechuic acid attenuates lipolysaccharide-induced acute lung injury. Inflammation..

[54] Rahimifard M, Maqbool F, Moeini-Nodeh S, Niaz K, Abdollahi M, Braidy N (2017). Targeting the TLR4 signaling pathway by polyphenols: A novel therapeutic strategy for neuroinflammation. Ageing Res Rev.

[55] Vanlangenakker N, Vanden Berghe T, Vandenabeele P (2012). Many stimuli pull the necrotic trigger, an overview. Cell Death Differ.

[56] Hildebrand JM, Tanzer MC, Lucet IS, Young SN, Spall SK, Sharma P, Silke J (2014). Activation of the pseudokinase MLKL unleashes the four-helix bundle domain to induce membrane localization and necroptotic cell death. P Nat Acad Sci USA.

[57] Fan H, Tang HB, Shan LQ, Liu SC, Huang DG, Chen X, et al. Quercetin prevents necroptosis of oligodendrocytes by inhibiting macrophages/microglia polarization to M1 phenotype after spinal cord injury in rats. J Neuroinflamm. 2019;16(1). 10.1186/s12974-019-1613-2. 10.1186/s12974-019-1613-2 PMC6839267 31699098

[58] Liu L, Liu Y, Cheng X, Qiao X (2021). The alleviative effects of quercetin on cadmium-induced necroptosis via inhibition ROS/iNOS/NF-kappaB pathway in the chicken brain. Biol Trace Elem Res.

[59] Bergsbaken T, Fink SL, Cookson BT (2009). Pyroptosis: host cell death and inflammation. Nat Rev Microbiol.

[60] Shi J, Zhao Y, Wang K, Shi X, Wang Y, Huang H, Shao F (2015). Cleavage of GSDMD by inflammatory caspases determines pyroptotic cell death. Nature..

[61] Zhang F, Liu T, Huang HC, Zhao YY, He M, Yuan W, Li L, Li J, Wu DM, Xu Y (2022). Activation of pyroptosis and ferroptosis is involved in radiation-induced intestinal injury in mice. Biochem Biophys Res Commun.

[62] Liu F, Wang XJ, Cui Y, Yin Y, Qiu D, Li SL, et al. Apple Polyphenols extract (APE) alleviated dextran sulfate sodium induced acute ulcerative colitis and accompanying neuroinflammation via inhibition of apoptosis and pyroptosis. Foods. 2021;10(11). 10.3390/foods10112711. 10.3390/foods10112711 PMC8619666 34828992

[63] Luo X, Bao X, Weng X, Bai X, Feng Y, Huang J (2022). The protective effect of quercetin on macrophage pyroptosis via TLR2/Myd88/NF-kappaB and ROS/AMPK pathway. Life Sci.

