# Supplementary material for: Protocatechuic acid and quercetin attenuate ETEC-caused IPEC-1 cell inflammation and injury associated with inhibition of necroptosis and pyroptosis signaling pathways
Source: J Anim Sci Biotechnol. 2023 Feb 1;14:5. doi: 10.1186/s40104-022-00816-x (PMC9890695; doi:10.1186/s40104-022-00816-x)
Supplement: Supplementary file 1 — Additional file 1. Primers used for real-time PCR analyses. [file 40104_2022_816_MOESM1_ESM.docx]

**Table 1** Primer sequences used for real-time PCR

| Gene | Forward (5'→3') | Reverse (5'→3') |
| --- | --- | --- |
| *LBP* | GAACACAGCCGAATGGTCTAC | GGAAGGAGTTGGTGGTCAGT |
| *TLR4* | TCAGTTCTCACCTTCCTCCTG | GTTCATTCCTCACCCAGTCTTC |
| *IRAK1* | CAAGGCAGGTCAGGTTTCGT | TTCGTGGGGCGTGTAGTGT |
| *MD2* | TGCAATTCCTCTGATGCAAG | CCACCATATTCTCGGCAAAT |
| *CD14* | CGTTTGTGGAGCCTGGAAG | TGCGGATGCGTGAAGTTG |
| *TNF-α* | TCCARATGGCAGAGTGGGTATG | AGCTGGTTGTCTTTCAGCTTCAC |
| *IL-6* | ARAGGTGATGCCACCTCAGAC | TCTGCCAGTACCTCCTTGCT |
| *IL-8* | ACAGCAGTARACARACARACARAG | GACCAGCACAGGARATGAG |
| *β-actin* | TGCGGGACATCARAGGAGARAG | AGTTGARAGGTGGTCTCGTGG |

TLR4, toll-like receptor; IRAK1, IL-1 receptor-associated kinase 1; LBP, LPS binding protein; MD2, myeloid differentiation factor-2; CD14, cluster differentiation factor-14; TNF-α, tumor necrosis factor-α; IL-6, interleukin -6; IL-8, interleukin -6; IL-18, interleukin-18
