# Supplementary material for: Protocatechuic acid and quercetin attenuate ETEC-caused IPEC-1 cell inflammation and injury associated with inhibition of necroptosis and pyroptosis signaling pathways
Source: J Anim Sci Biotechnol. 2023 Feb 1;14:5. doi: 10.1186/s40104-022-00816-x (PMC9890695; doi:10.1186/s40104-022-00816-x)
Supplement: Supplementary file 2 — Additional file 2. Antibacterial effects of PCA and Que on ETEC K88 growth. After inoculating ETEC K88 into LB medium, 40 μmol/L PCA or 10 μmol/L Que or PBS were added into the holes to incubate for 24 h at 37 °C. [file 40104_2022_816_MOESM2_ESM.docx]

**Supplemental figure 1**

Effects of PCA and Que on the bacterial growth.


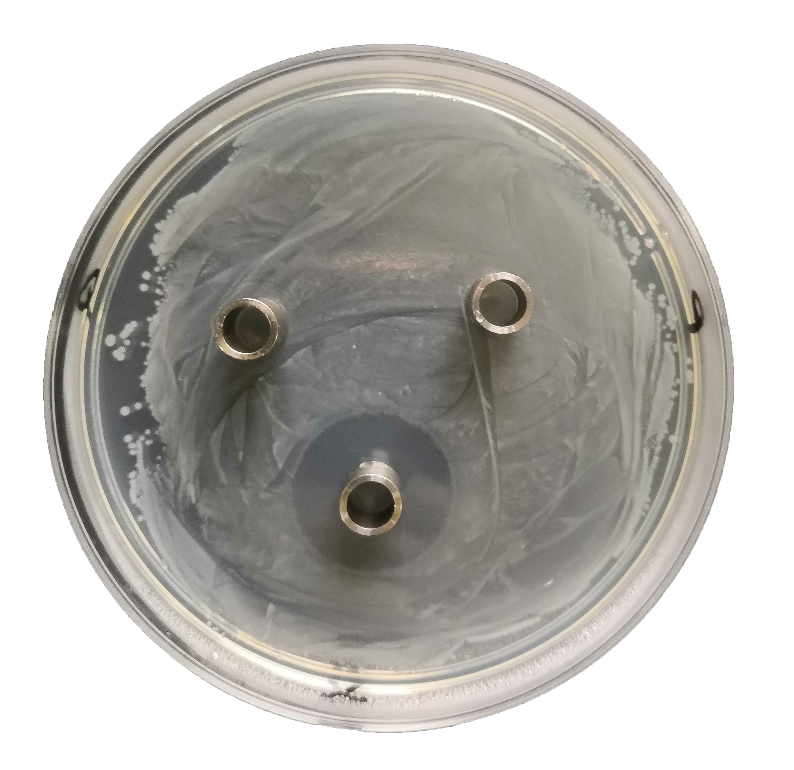


**Que 10 μmol/mL**

**PCA 40 μmol/mL**

**Antibiotic**
