# Supplementary figures and images for: Protocatechuic acid and quercetin attenuate ETEC-caused IPEC-1 cell inflammation and injury associated with inhibition of necroptosis and pyroptosis signaling pathways
Source: J Anim Sci Biotechnol. 2023 Feb 1;14:5. doi: 10.1186/s40104-022-00816-x (PMC9890695; doi:10.1186/s40104-022-00816-x)

Supplemental figure 2


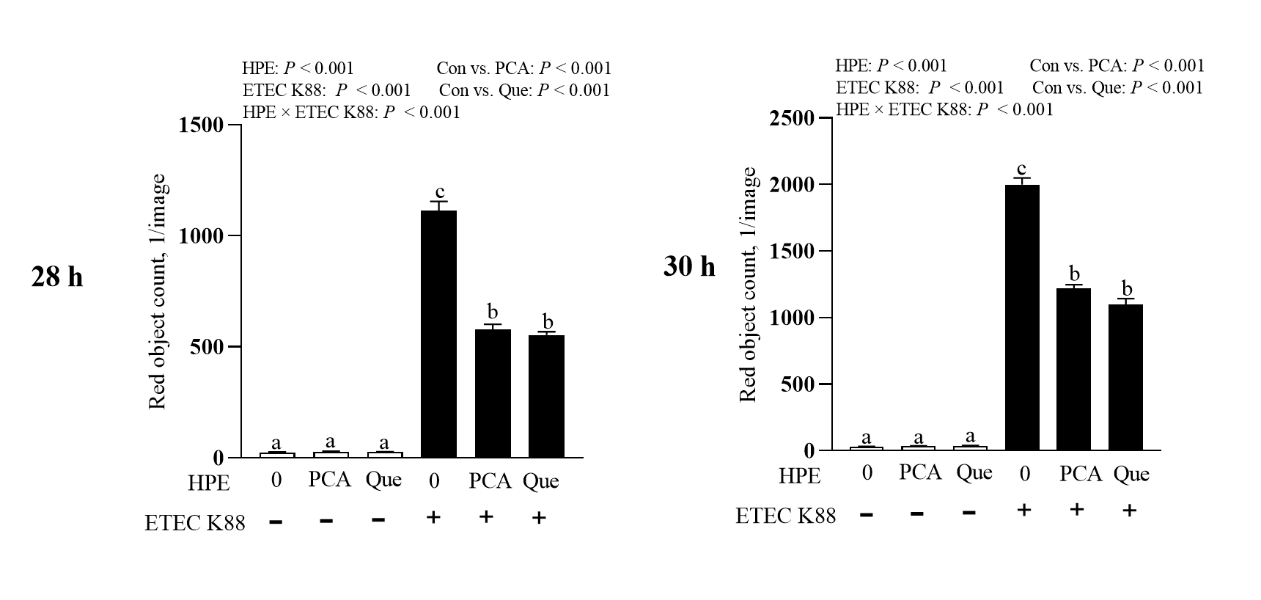

Supplement: Supplementary file 4 — Additional file 4. Effects of PCA and Que on cell necrosis in IPEC-1 cells infected with ETEC K88 at 28 and 30 h (4 and 6 h after ETEC K88 infection, respectively). Cells were pre-treated with 40 μmol/L PCA or 10 μmol/L Que for 24 h and then infected with or without 1 × 10^8^ ETEC K88/mL for 6 h. Values are means ± SE, n = 6. a–cDifferent letters represent a significant difference, P < 0.05. IPEC-1, intestinal porcine epithelial cell 1. [file 40104_2022_816_MOESM4_ESM.docx]
